# One step beyond a broad molecular phylogenetic analysis: Species delimitation of *Adenomera marmorata* Steindachner, 1867 (Anura: Leptodactylidae)

**DOI:** 10.1371/journal.pone.0229324

**Published:** 2020-02-21

**Authors:** Carla S. Cassini, Pedro P. G. Taucce, Thiago R. de Carvalho, Antoine Fouquet, Mirco Solé, Célio F. B. Haddad, Paulo C. A. Garcia

**Affiliations:** 1 Departamento de Ciências Biológicas, Universidade Estadual de Santa Cruz, Ilhéus, Bahia, Brazil; 2 Departamento de Biodiversidade, Instituto de Biociências, Centro de Aquicultura (CAUNESP), Universidade Estadual Paulista ‘Júlio de Mesquita Filho’, Rio Claro, São Paulo, Brazil; 3 Laboratoire Évolution et Diversité Biologique, UMR5174, CNRS-UPS-IRD, Bâtiment, France; 4 Departamento de Zoologia, Instituto de Ciências Biológicas, Universidade Federal de Minas Gerais, Belo Horizonte, Minas Gerais, Brazil; Universitat Trier, GERMANY

## Abstract

Taxonomists always have had intense discussions about how species should be delimited and recently many studies have used integrative approaches by combining molecular, morphological, and bioacoustic data. Although these studies are paramount for understanding species diversity, few of them actually formalize species delimitations to the final step of nomenclatural acts. Historically, the Neotropical frog genus *Adenomera* has been considered as a difficult taxonomic group because it comprises many morphologically similar species exhibiting high levels of intraspecific polymorphism. A recent work using molecular data shed light on the phylogenetic relationships within the genus and identified several lineages that may correspond to undescribed species but did not delimit species boundaries. In the Atlantic Forest, a clade formed by *A*. *marmorata* and two putative species (*Adenomera* sp. J and *Adenomera* sp. K) were identified. In this paper, we combine morphological, acoustic, and molecular data in order to evaluate species limits within this Atlantic Forest *Adenomera* clade. We provide a redescription of *A*. *marmorata* and restrict its type locality to the Tijuca Massif, in the Municipality of Rio de Janeiro, Brazil. Our results do not support *A*. *marmorata* and the two candidate species as diagnosable distinct species. Therefore *A*. *marmorata* corresponds to a species with pronounced morphological and acoustic variation in the genus and a complex phylogeographic structure.

## Introduction

The recent advent of integrative taxonomy (term formally introduced by [1] and [2]) has led to an intensive discussion about how scientists should delimit species (e.g. [3–5]). Consequently, many studies have also emerged in the past few years using integrative taxonomy protocols to delimit species by applying replicable and testable criteria (see [6] for a survey). Molecular analyses allow researchers to estimate biodiversity levels more quickly. Therefore, it is paramount to give a step further and formally describe the biodiversity in order to support a wide range of biological research (e.g., studies of evolution, ecology, and conservation) and to subsidize conservation polices.

Among vertebrates, lepidosaurians and amphibians are the most studied taxa through an integrative framework [6]. Nevertheless, a recent assessment found that more than 50% of those studies recognized the existence of new species without formally describing them [6], which means that even though species have been discovered, species descriptions have been carried out only by a parcel of the systematists and taxonomists in these taxonomic groups.

The anuran genus *Adenomera* Steindachner, 1867 occurs in most part of South America east of the Andes [7]. These small leaf-litter frogs have been neglected by taxonomists because they are usually associated with morphologically cryptic species complexes (e.g. [8–10]). *Adenomera* currently comprises 21species [11] and due to its complex taxonomy, most of them were described (or revalidated) with both morphological and acoustical data assessed to corroborate the taxonomic decisions (e.g. [12–14]). Nevertheless, molecular data has been used to delimit just a few species in the genus.

Fouquet *et al*. [7] provided the first comprehensive study using molecular data to delimit candidate species in *Adenomera*. These authors sampled across the distribution of the genus and estimated the existence of at least 20 unnamed (confirmed and unconfirmed) candidate species throughout Amazonia, Atlantic Forest, Caatinga, Cerrado, and Chaco domains [7]. This study also evaluated other lines of evidence to assess the candidate species status, such as advertisement calls, and morphology of adults and tadpoles, mostly from the literature. In the Altantic Forest, they identified a clade formed by *A*. *marmorata* and two putative species (*Adenomera* sp. J and *Adenomera* sp. K) considered as confirmed on the basis of the concordant mtDNA and nuDNA data but meager acoustic and morphological data suggesting phenotypic divergence. Despite the important contribution of Fouquet *et al*. [7] to the diversity patterns in *Adenomera*, it was beyond the scope of that study to formally describe candidate species. Therefore, a formal taxonomic review based on an integrated approach is still needed to validate the species hypothesis formerly proposed by Fouquet *et al*. [7] as confirmed candidate species (hereafter CCS). To avoid confusion between the nominal species *Adenomera marmorata* and the CCS *Adenomera marmorata* as defined by Fouquet *et al*. [7], hereafter we will refer to the CCSs *Adenomera marmorata*, *Adenomera* sp. J, *Adenomera* sp. K as CCS Ma, CCS J and CCS K, respectively.

Assigning populations to *Adenomera marmorata* is a well-known problem due to the high level of inter and intrapopulation polymorphism, the lack of an exact type locality and a brief original description. Additionally, there are few available synonyms under the name *Adenomera marmorata* [11]. Therefore, it is often difficult for herpetologists to confidently associate populations with species names. Given that *A*. *marmorata* is the type species of the genus, clarifying its taxonomical boundaries should be relevant to understand species’ identities in the genus, especially those distributed across the Atlantic Forest domain. Notwithstanding, taxonomical contributions for the genus *Adenomera* within this domain are seldom published. Before the recent description of *A*. *kweti* [15], the most recent taxonomical contribution for the genus in the Atlantic Forest domain was published a decade ago [16].

Herein we aim to delimit the species *Adenomera marmorata* by evaluating the taxonomic status of the confirmed candidate species (CCS) defined by Fouquet *et al*. [7] (herein referred as CCS J and CCS K), using an integrative taxonomy protocol based on a comprehensive dataset of phenotypic (morphology and calls) and molecular data.

### Taxonomic history

The original description of *Adenomera marmorata* is brief and based on a single specimen. The description was the first for the genus and largely agrees with any currently described *Adenomera* species [17]. Nevertheless, some valuable taxonomic characters were reported later and used in the taxonomy of the genus, as the expanded toe tips present in the specimen ([18]; and subsequent descriptions of new species). Steindachner designated the species’ type locality as “Brasilien” (Brazil). However, the holotype was collected during the “Novara Reise” (Novara Expedition), which visited several localities within the municipality of Rio de Janeiro, state of Rio de Janeiro, and neighboring municipalities [19, 20]. Some authors agree that the holotype was collected in Rio de Janeiro or its surroundings [21, 22].

Adolpho Lutz [23] described one *Adenomera* species, *Leptodactylus trivittatus*, based on specimens from “Campo Bello” and “Alto da Serra de Cubatão”, currently the municipalities of Itatiaia, state of Rio de Janeiro, and Santo André, state of São Paulo, respectively [21] The species name was given based on one dorsal and two dorsolateral brick red stripes present in the specimens. At the time, Lutz did not relate *L*. *trivittatus* to *A*. *marmorata*, but to *L*. *nanus* [24].

Bertha Lutz [25] suggested that *L*. *trivittatus*, described by A. Lutz [23], was a junior synonym of *L*. *nanus*, considering that the series of *L*. *trivittatus* had one of the color morphotypes of *L*. *nanus*. A few years later, Cochran [26] redescribed *Adenomera marmorata* (as *Leptodactylus marmoratus*) and considered both *L*. *nanus* and *L*. *trivittatus* as their synonyms. This author argued that the coloration pattern of *A*. *marmorata* was highly variable and the three species actually comprised color morphotypes among populations of the same species.

Heyer [18] kept *L*. *nanus* and *L*. *trivittatus* in the synonymy of *Adenomera marmorata* and designed lectotypes for both species. With respect to *L*. *trivittatus*, the chosen specimen was a juvenile, because it was the only among the syntypes possessing the striped pattern that was used as evidence to give name to the species. However, Heyer [18] was not clear when fixed the type locality to “Campo Bello, Alto da Serra de Cubatão, Brasil”, since the two localities are different places, in different mountainous complexes (Serra da Mantiqueira and Serra do Mar mountain ranges, respectively). Cochran [26] examined the specimen defined as the lectotype of *L*. *trivittatus* and specified the collection site of the specimen as “Montserrat, Campo Bello, Rio de Janeiro”, which is currently the headquarters of the Itatiaia National Park, municipality of Itatiaia. More recently, Kwet [13] revalidated *L*. *nanus* and kept *L*. *trivittatus* in the synonymy of *A*. *marmorata*.

Fouquet *et al*. [7] assigned to the CCS *Adenomera* sp. J (CCS J) the populations from Santo André, state of São Paulo ("Alto da Serra de Cubatão”, [23]), and the populations from Itatiaia, state of Rio de Janeiro (“Campo Bello”; [25]) to *Adenomera* sp. K (CCS K) based on geographical distribution data. The junior synonym *Leptodactylus trivittatus*, if valid, should correspond to the population from the municipality of Itatiaia, state of Rio de Janeiro, therefore the CCS *Adenomera* sp. K (CCS K) [7].

## Material and methods

### Species delimitation

We conducted field expeditions to previously known occurrence localities of *Adenomera* belonging to CCSs Ma, J and K, and additional localities in these regions in an attempt to increase molecular and phenotypic data. All recorded males had also been genotyped (S1 Table), except a few males recorded only (no sequence) from localities where only one clade was found. As envisaged by the unified concept of species [27], species should be delimited based on the following criteria: 1) cladogram topology and 2) identification of at least one diagnostic phenotypic evidence that supports the independency of evolutionary lineages from its sister clade.

### Molecular analyses

#### Taxon sampling

We obtained new DNA sequences from 149 tissue samples from CCS Ma (n = 73), CCS J (n = 51), and CCS K (n = 25). We also obtained sequences from two tissue samples of *A*. *ajurauna* [28] from the municipality of Santo André, state of São Paulo, because they occur syntopically with *A*. *marmorata* and two of *A*. *thomei* [22] from the municipality of Itatiaia, state of Rio de Janeiro, because they occur sympatrically with the CCS K. We also used sequences analyzed by Fouquet *et al*. [7] available in GenBank.

We used as outgroup all remaining *Adenomera* candidate species sequences downloaded from GenBank, except for *A*. *coca*, which has been recovered nested within A. hylaedactyla clade [7]. For the outgroup we chose sequences from one (if just one sequence was available) to four terminals of each candidate species, depending on the number of available sequences of each candidate species subclades [7]. Genera other than *Adenomera* sampled as outgroup consisted of sequences obtained from GenBank from one *Hydrolaetare caparu* [29], one *Lithodytes lineatus* [30], and one *Leptodactylus rhodomystax* [31].

#### Laboratory methods

Whole cellular DNA was extracted from ethanol-preserved muscle (primarily) or liver tissue samples following Lyra *et al*. [32] or using a DNeasy Qiagen kit following manufacturer’s protocols. PCR amplification was carried out using Taq DNA Polymerase Master Mix (Ampliqon S/A, Denmark) and Axygen Maxygene thermocyclers. We targeted three mitochondrial (Cytochrome c oxidase subunit 1 [COI], Cytochrome b [CYTB], and 16S ribosomal DNA [16S]) and two nuclear (Proopiomelanocortin C [POMC] and Recombination activating gene exon1 [RAG]) gene fragments (primer pairs detailed in Table 1). The standard PCR program consisted in an initial denaturing step of 2 minutes at 94°C, 35–40 cycles of 30 seconds at 94°C, 30 seconds at 48–56°C, and 2 minutes at 72°C, followed by a final extension step of 10 minutes at 72°C. PCR non-purified products were sent to Macrogen Inc. (South Korea) where they conducted purification and sequencing in an ABI 3730XL sequencer.

**Table 1:** Gene fragments and respective primer pairs used in DNA amplification.

Primer		Gene	Sequence
dgLCO1490 [33]	F	COI	GGTCAACAAAATCATAAAGAYATYGG
dgHCO2198 [33]	R	COI	TAAACTTCAGGGTGACCAAARAAYCA
Cytb2 [34]	F	Cytb	AAACTGCAGCCCCTCAGAAATGATATTTGTCCTCA
MVZ15 [35]	F	Cytb	GAACTAATGGCCCACACWWTACGNAA
CbR2 [36]	R	Cytb	GTGAAGTTRTCYGGGTCYCC
16SAR [37]	F	16S	CGCCTGTTTATCAAAAACAT
16SWilk2 [38]	R	16S	GACCTGGATTACTCCGGTCTGA
MartFL1 [39]	F	RAG	AGCTGGAGYCARTAYCAYAARATG
Ad2R [36]	R	RAG	ATTGGCTCTCCATGTTTCATAG
POMC 1 [40]	F	POMC	GAATGTATYAAAGMMTGCAAGATGGWCCT
POMC 2 [40]	F	POMC	TCTGCMGARTCWCCYGTGTTTCC
POMC 3 [40]	R	POMC	TAYTGRCCCTTYTTGTGGGCRTT
POMC 4 [40]	R	POMC	TGGCATTYTTGAAAAGAGTCAT

#### Alignment, partition schemes, and model selection

We performed alignment using MAFFT v. 7.273 [41] with the FFT-NS-i algorithm, except for the 16S gene fragment, for which we used the E-INS-i algorithm. This strategy is applicable for regions with conserved domains surrounded by nonalignable regions, such as rDNA. For both algorithms we used 1000 as a maximum for iterative refinements.

We conducted the search for the best partition scheme and best fitting molecular model using PartitionFinder 1.1.1 [42] with the Corrected Akaike Information Criterion (AICc; [43]) and considering each codon as a separate partition.

#### Phylogenetic analyses, haplotype network, and genetic distance

We used both Maximum Likelihood (ML) and Bayesian Inference (BI, concatenated locus approach) for constructing the phylogenetic trees. We conducted ML analysis in RAxML v. 8.2.10 [44], with 100 runs for tree search and 1000 non-parametric bootstrap replicates. We constructed BI trees in MrBayes [45] using two independent runs of 2.0 x 10^7^ generations, starting with random trees and four Markov chains (one cold), sampled every 2000 generations. We discarded 25% of generations and trees as burn-in and performed the run with unlinked character state frequencies, substitution rates of GTR model, gamma shape parameters, and proportion of invariable sites between partitions.

We examined if candidate species display signs of reproductive isolation by analyzing nuclear DNA (nDNA), allele sharing, and network cohesion. To determine the most probable alleles for individuals heterozygous for nDNA sequences we used gametic phases reconstructed through the algorithm implemented in phaSe 2.1.1 software [46], which were interconverted to fasta format using SeqPHASE web tool [47]. Using these alignments we computed statistical parsimony networks using TCS [48] method.

We computed uncorrected pairwise *p-*distances using R version 3.6.0 [49] with the packages APE version 5.0 [50] and SPIDER version 1.5.0 [51]. In order to avoid alignment effect, we ignored sites within missing data (*pairwise*.*deletion* = TRUE).

### Phenotypic analyses: Morphology and advertisement call

We genotyped all collected and recorded specimens. Thus, we could reliably associate morphological and acoustic data for all specimens of candidate species from the *A*. *marmorata* clade (*sensu* Fouquet *et al*. [7]). The morphological analyses followed characters described by Heyer [18], Heyer *et al*. [52], and Duellman [53]. For the morphometric variation we used SVL (snout-vent length) and, additionally, for the holotype description, we measured HL (head length), HW (head width), ED (eye diameter), TD (tympanum diameter), END (eye to nostril distance), IND (internarial .distance), IOD (interorbital distance), THL (thigh length), SL (shank length), and TAL (tarsal length). All measurements were taken to the nearest 0.05 mm with digital calipers under a stereomicroscope. Material examined is given in S1 Table.

Calls were recorded using digital recorders (Marantz PMD 670, PMD 671, and Tascam DR-40) set at a sampling rate of 44.1 kHz and sample size of 16 bits, and Sennheiser K6/ME66 and K6/ME67 unidirectional microphones. Recordings were stored as mono-channel uncompressed WAVE files. Sound files were deposited in the acoustic repositories of AAG-UFU and CFBH collections (see S2 Table). Voucher specimens are housed in the following Brazilian collections: Coleção de Anfíbios do Centro de Coleções taxonômicas da Universidade Federal de Minas Gerais (UFMG), Belo Horizonte, MG, and Coleção de Anfíbios Célio F. B. Haddad (CFBH), at Universidade Estadual Paulista, Rio Claro, SP.

Acoustic analysis was conducted in Soundruler [54], built as a package interfacing with Matlab scripts [55] through automated procedures that allow for unbiased quantification of acoustic traits. Grand means (and corresponding standard deviations) were obtained from averages of each male recorded. Parameters were set as follows: FFT size = 1024 samples, FFT overlap = 90% or 99%, window type = Hanning, contrast = 70%. Settings for automated recognition were (in sample sizes): detection (smoothing = 500, resolution = 1); delineation (smooth factor = 1, smoothing = 13, and resolution = 1). In a few cases, raw values for the fundamental frequency exceeded those of the dominant frequency of a given call. This is because the automated analysis quantifies those frequency traits differently and may reflect minor sampling resolution errors in the measurements up to ± 50 Hz. A 1000-Hz high-pass filter was applied to sound files in Soundruler prior to conducting the acoustic analyses to reduce background noise. Call rate was quantified manually in Audacity 2.1.1 [56]. Acoustic terminology and definitions are given in S3 Table.

Sound figures were produced using seewave 2.1.0 [57] and tuneR 1.3.2 [58] in R 3.6.0 [49]. Settings for sound figures were: window Hanning, FFT size = 256 samples, FFT overlap = 90%; the intensity of frequency components is indicated by their darkness in a relative 36-dB scale.

In order to assess the degree of acoustic differentiation among candidate species, we performed a Principal Component Analysis (PCA) in R 3.6.0 [49] with the function *prcomp*. We visualized the PCA using the R packages ggbiplot [59] and factoextra [60].

## Results

### Alignment, partition scheme, and model selection

We obtained a final dataset of 3322 bp of aligned DNA sequences from nuclear and mitochondrial genes: partial 16S rRNA (561 bp), partial COI (657 bp), partial CYTB (666 bp), partial POMC (608 bp), and partial RAG1 (830 bp). The best-fit partition scheme comprised 11 partitions. The partitions and respective best fitting nucleotide substitution models used in the BI analysis are listed in Table 2. For the ML analysis we used the GTR model with a γ-distribution for all partitions, since RAxML does not support estimating different substitution models for different partitions.

**Table 2:** Model selection. Best partition scheme and respective best fitting molecular models used in phylogenetic analyses. Numbers following gene names correspond to the codon position.

Partition	Model
16S	GTR+I+Γ
COI 1	GTR+I+Γ
COI 2	HKY+I
COI 3	GTR+Γ
CYTB 1	SYM+I+Γ
CYTB 2	GTR+I+Γ
CYTB 3	GTR+Γ
POMC 1 + RAG 1	GTR+I+Γ
POMC 2	GTR+Γ
POMC 3 + RAG 3	GTR+I+Γ
RAG 2	HKY+I+Γ

### Phylogenetic analyses and genetic distance

**Fig 1:**
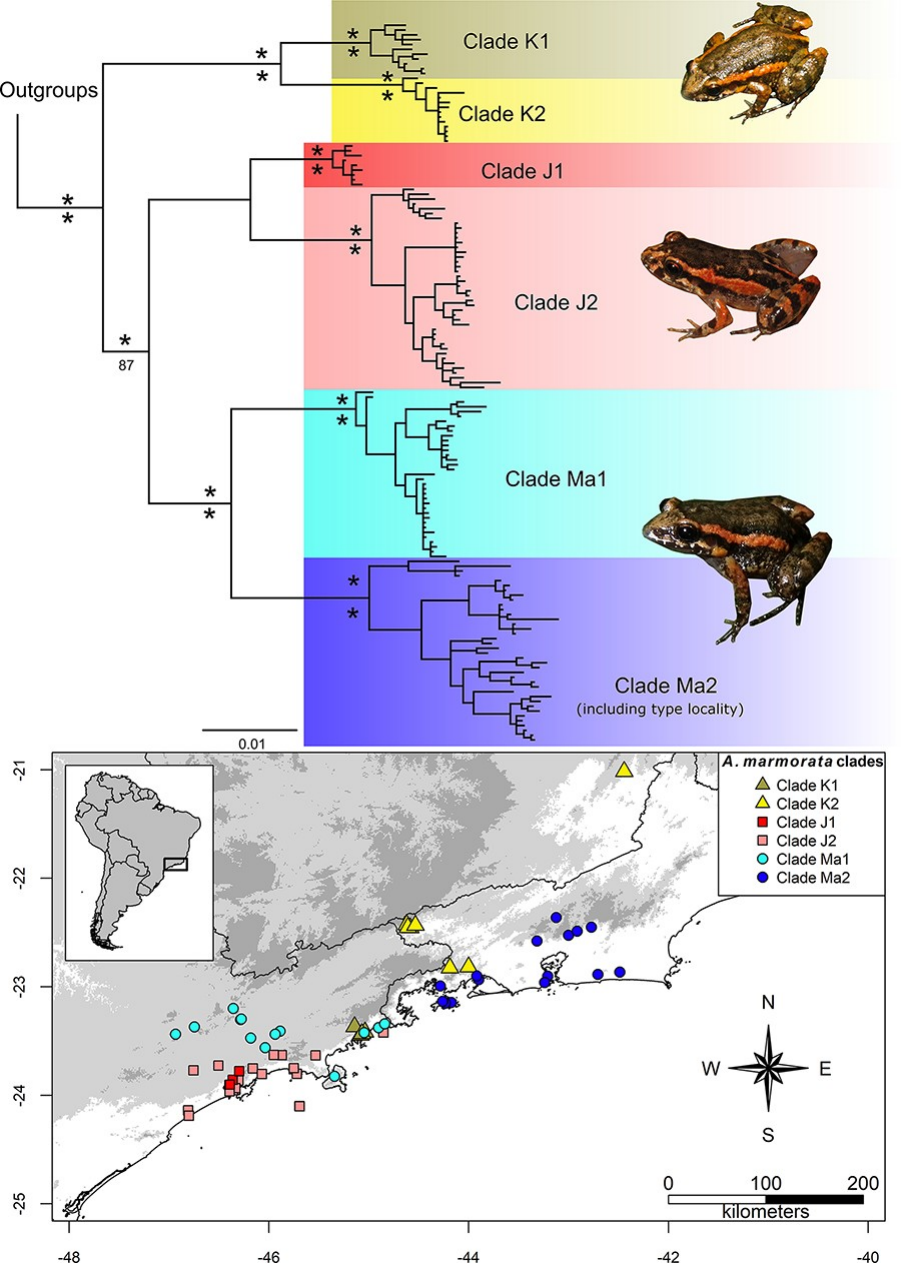
Bayesian inference and genetic samples. 50% majority rule consensus tree from Bayesian inference analysis of concatenated nuclear (POMC and RAG) and mitochondrial (COI, CYTB, and 16S) genes, and map containing sampled localities for molecular analyses. Numbers above branches indicate Bayesian posterior probabilities (pp) and numbers below branches indicate non-parametrical bootstrap support. Asterisks indicate clades fully supported (1.0 [pp] or 1000 [bootstrap]). Colors of the clades match colors of the localities.

The BI and ML analyses yielded the same topology (Fig 1, S1 Fig and S2 Fig). We recovered all nominal and candidate *Adenomera* species as monophyletic with high support in both analyses. We will not further comment about the relationships between outgroup species because this is beyond the scope of the present paper.

We recovered CCS K as the sister group of “CCS J + CCS Ma”. The CCS K is structured in two well-supported clades (K1 and K2). The clade K1 contains specimens from the northern coast of São Paulo state (our samples are restricted to the main district of the municipality of Ubatuba and São Luis do Paraitinga, state of São Paulo); the clade K2 contains specimens from Minas Gerais and southern inland Rio de Janeiro state, including the type locality of *Leptodactylus trivittatus*.

The CCS J lies in the central and northern coastal areas of São Paulo state and adjacent Serra do Mar, and is also structured in two major clades, J1 and J2 clades, both well-supported. Specimens from clade J1 are geographically circumscribed within the range of calde J2; i.e. specimens from clade J1 are restricted to the municipalities of Cubatão and Santo André, other individuals from these localities were also recovered in clade J2.

The CCS Ma includes two main clades, Ma1 and Ma2, both well-supported. The clade Ma1 lies primarily in the inland eastern São Paulo state, reaching its northern coast (including Picinguaba, a locality within the municipal limits of Ubatuba); the clade Ma2 lies in the southern coast of Rio de Janeiro state and adjacent Serra do Mar, including the presumed type locality of the species *Adenomera marmorata*.

The uncorrected pairwise distances between CCS J and its sister clade, CCS Ma, varies from 2.8 to 5.8% (16S, Table 3) and 4.9 to 12.2% (COI). Genetic distances between CCS J and CCS Ks varies from 2.6 to 4.1% (16S) and 7.0 to 11.9% (COI). Genetic distances between CCS K and CCS Ma varies 2.8 to 6.7% (16S) and 6.2 to 10.8% (COI).

**Table 3:** Partial 16s (light grey) and partial COI (dark grey) average uncorrected pairwise *p*-distances within and between lineages. Distances among CCSs Ma, J and K are presented in bold. Data are shown as min—max when applicable.

	Within CCS *p*-distance		Between CCS *p*-distance								
	16S (*n*)	COI (*n*)	1	2	3	4	5	6	7	8	9
1. *A*. *ajurauna*	0.0–1.8 (3)	0.0–6.7 (7)		12.4–15.7	11.6–14.6	11.2–13.6	7.3–13.6	10.1–12.9	12.2–14.9	7.7–14.2	10.4–12.6
2. *A*. *araucaria*	––(1)	0.0–9.8 (9)	5.5–6.4		10.6–16.7	11.4–15.0	12.4–16.1	11.5–14.8	9.8–12.1	11.7–19.1	10.6–15.6
3. *A*. *bokermanni*	––(1)	6.5 (2)	9.6–10.2	7.0		8.7–10.0	11.3–14.6	13.2–17.0	11.1–14.4	10.7–17.5	7.9–10.7
4. *A*. *engelsi*	––(1)	0.5–3.6 (4)	6.9–7.7	6.3	7.1		11.6–14.8	11.3–15.3	10.5–12.4	10.9–15.4	6.7–8.4
**5. CCS J**	0.0–3.0 (13)	0.0–6.8 (51)	3.9–4.7	5.5–6.2	7.3–8.1	4.4–6.0		**7.0–11.9**	12.6–15.9	**4.9–12.2**	10.3–13.2
**6. CCS K**	0.0–2.0 (8)	0.0–5.8 (25)	3.7–5.1	5.0–6.2	8.3–8.9	5.2–7.3	**2.6–4.1**		11.0–14.9	**6.2–10.8**	11.8–14.9
7. *A*. *kweti*	––(1)	0.0–7.3 (8)	5.9–7.4	4.6	5.5	5.6–5.6	5.0–5.6	5.9–8.0		9.6–14.7	9.6–13.0
**8. CCS Ma**	0.0–4.8 (18)	0.0–8.2 (70)	4.5–6.8	5.0–6.4	8.3–9.1	5.4–6.7	**2.8–5.8**	**2.8–6.7**	5.5–6.9		9.9–15.9
9. *A*. *nana*	––(1)	3.6–4.6 (3)	6.3–7.2	5.0	3.6	4.6–4.6	4.8–5.4	5.4–7.0	4.8	4.8–5.6	

**Fig 2:**
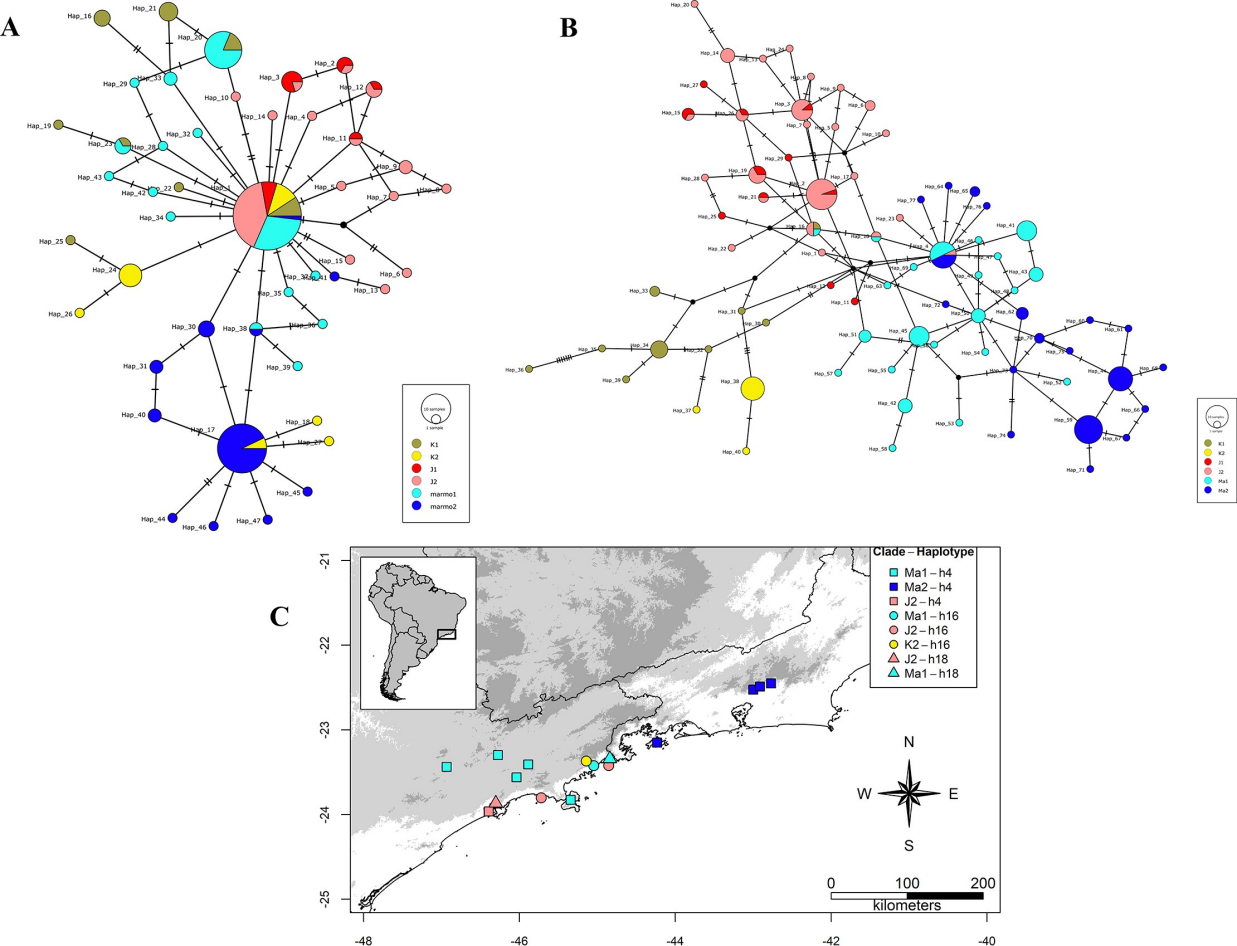
Statistical parsimony networks of phased nuclear loci for CCS Ma, CCS J and CCS K. **(**A) POMC; (B) fragment RAG1; (C) map containing RAG1 sampled localities for CCSs Ma, J and K. Haplotypes are shown as circles proportional in size to haplotype frequency.

We recovered 47 haplotypes for POMC and 77 haplotypes for RAG fragments. The TCS network for POMC gene revealed that the most abundant haplotypes were shared among all CCSs (J, K, and Ma) and the second most abundant haplotype (h17) was shared between CCS K (clade K2) and CCS Ma (clade Ma2). Addittionally, CCS K (clade K1) and CCS Ma (clade Ma1) lineages share two haplotypes (h20 and h23; Fig 2). In the corresponding network for the RAG fragment, there is essentially no haplotype sharing among lineages, except for three haplotypes (h4, h16, and h18). All CCSs share the haplotype h16 (clades Ma1, J2, and K2); CCS K and CCS Ma share the haplotype h4 (clades Ma1, Ma2, and J2); and CCS J and CCS Ma share haplotype h18 (clades Ma1 and J2; Fig 2).

### Phenotypic analyses: Morphology and advertisement call

**Fig 3:**
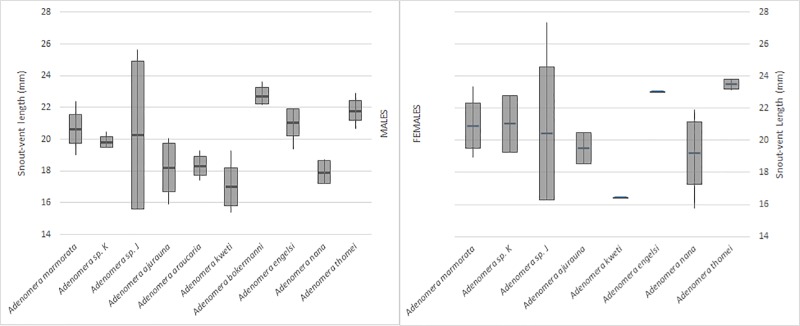
Variation of Snout-Vent Length among CCS Ma, CCS J, CCS K and other *Adenomera* species. Male (left) and female (right) specimens.

**Fig 4:**
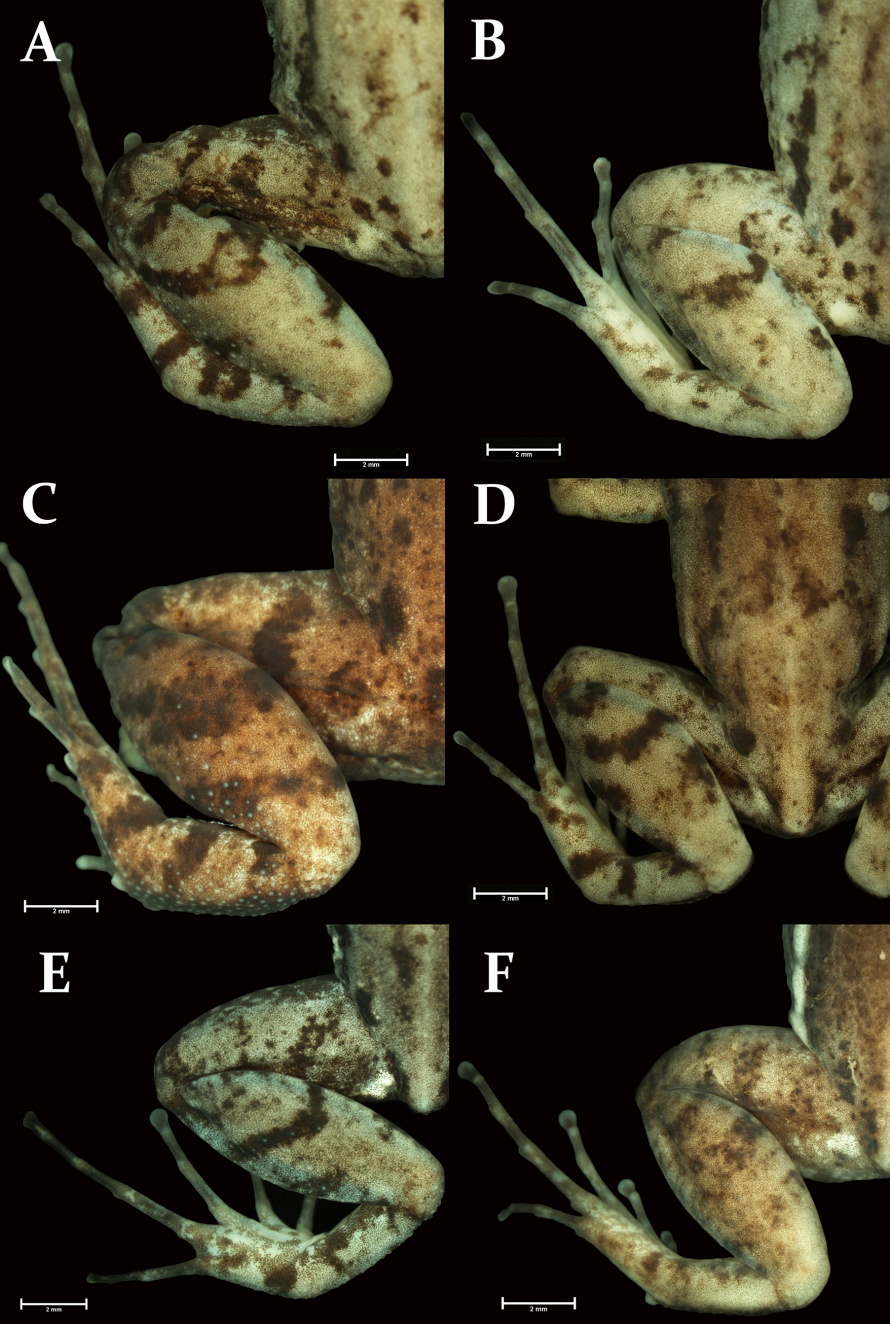
Morphological variation regarding the presence or absence of tibial tubercles. (A) CFBH 34402, CCS Ma, Rio de Janeiro, RJ; (B) CFBH 36131, CCS Ma, Nazaré Paulista, SP; (C) CFBH 17130, CCS J, Alcatrazes Island, SP; (D) CFBH 23923, CCS J, Santos, SP; (E) CFBH 36001, CCS K, Ubatuba, SP; (F) CFBH 444653, CCS K, Itatiaia, RJ.

Morphological variation among the CCSs J, K and Ma did not allow us to distinguish them from each other. The SVL completely overlapped among the three groups of specimens (Fig 3). Dorsolateral stripe was present in specimens in populations of all three CCSs. Tubercles on the dorsal surface of the tibia were present in CCS Ma and CCS K. Specimens from continental populations of CCS J did not have tubercles on the tibia. Nevertheless, specimens of CCS J from the Alcatrazes Island had tubercles on dorsal surface of the tibia similar to CCS Ma and CCS K (Fig 4). Specimens of CCS J from Alcatrazes Island are also larger (male SVL: 25.6 mm, n = 1; maximum female SVL: 27.3, n = 3) than those of continental populations of CCS J (maximum male SVL: 20.5 mm, n = 5; maximum female SVL: 19.4 mm, n = 9).

**Fig 5:**
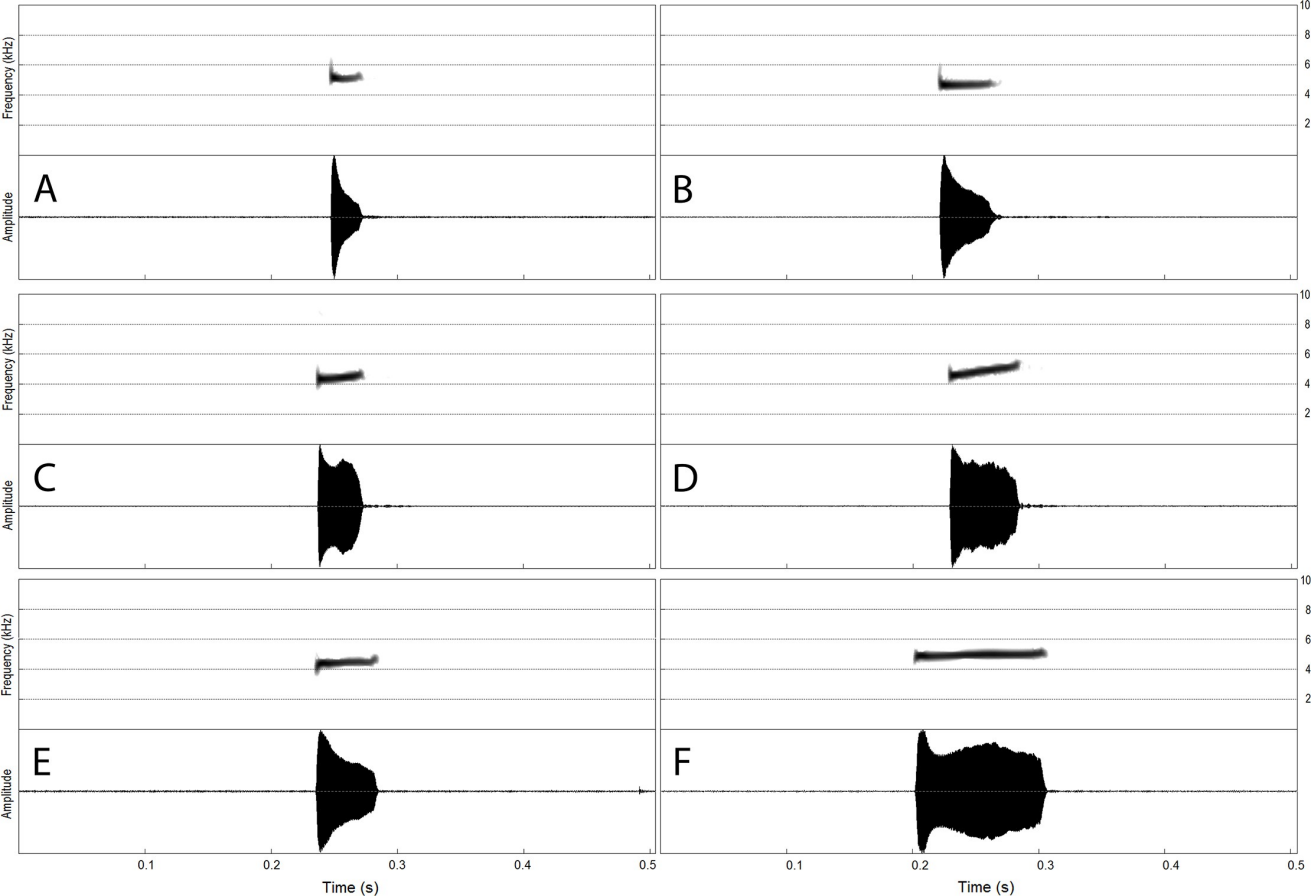
Advertisement call of the candidate species CCS Ma, CCS J and CCS K. Spectrogram (above) and oscillogram (bellow) of one call of (A) CCS Ma (clade Ma1), Tijuca Massif, Rio de Janeiro, RJ; (B) CCS Ma (clade Ma2), Nazaré Paulista, SP; (C) CCS K (clade K1), Itatiaia, RJ; (D) CCS K (clade K2), São Luís do Paraitinga, SP; (E) CCS J (clade J1), Mogi das Cruzes, SP; (F) CCS (clade J2), Santo André, SP.

We analyzed the advertisement calls of 51 individuals of CCS Ma, J, and K from 10 localities (municipalities of Guapimirim, Itatiaia, Maricá, Petrópolis, and Rio de Janeiro, state of Rio de Janeiro, and Mogi das Cruzes, Nazaré Paulista, Santo André, São Luís do Paraitinga, and Ubatuba, state of São Paulo), totaling 1951 calls. The advertisement calls of the three lineages showed similar structure: a single, nonpulsed note emitted at regular intervals, having or not a frequency upsweep along the note (Fig 5). The dominant frequency coincides with the fundamental harmonic. Although CCS J tended to have calls with shorter durations and higher in pitch than those CCS Ma and CCS K, the acoustic traits analyzed usually overlapped among the them (Table 4).

**Table 4:** Advertisement call traits of the candidate species CCS Ma (Ma1 and Ma2), CCS J (J1 and J2), and CCS K (K1 and K2). Data are given as mean ± SD (range); sample sizes as number of males recorded/number of quantified calls. Repetition rate was quantified once or twice for each male. Temporal call traits are given in milliseconds (ms), and frequency traits in Hertz (Hz).

Call traits	CCS Ma		CCS J		CCS K	
	Ma1 n = 2/76	Ma2 n = 8/269	J1 n = 14/665	J2 n = 3/148	K1 n = 10/205	K2 n = 14/598
TEMPORAL TRAITS						
Repetition rate (minute)	51.9 ± 10.6 (44.3–59.4)	32.9 ± 17.2 (14.4–66.4)	51.0 ± 10.0 (36.0–67.5)	44.2 ± 4.1 (39.6–47.4)	64.2 ± 15.3 (41.8–83.3)	45.4 ± 9.7 (30.6–63.4)
Call duration	40.9 ± 11.6 (28.3–51.5)	58.7 ± 14.5 (33.5–91.0)	29.0 ± 6.0 (21.0–45.0)	33.9 ± 5.1 (31.0–39.7)	58.0 ± 9.0 (40.0–77.0)	97.2 ± 6.6 (77.7–113.9)
Rise time (%)	6.2 ± 0.8 (4.2–9.6)	8.1 ± 5.1 (1.8–77.9)	10.5 ± 3.8 (4.8–27.8)	8.7 ± 2.3 (7.2–11.4)	6.2 ± 1.0 (3.1–24.2)	9.4 ± 8.0 (2.3–76.0)
Plateau duration	2.3 ± 0.8 (1.1–5.2)	3.1 ± 1.1 (0.3–16.1)	3.4 ± 1.2 (1.9–11.0)	3.0 ± 0.1 (2.8–3.1)	2.0 ± 1.6 (0.1–6.7)	6.2 ± 3.2 (0.4–35.5)
Attack duration	1.7 ± 0.8 (1.0–3.0)	3.0 ± 2.1 (0.7–33.9)	1.8 ± 0.9 (1.0–5.0)	1.7 ± 0.5 (1.2–2.2)	2.7 ± 0.7 (1.2–14.7)	6.6 ± 6.7 (1.3–65.8)
Decay duration	36.9 ± 10.0 (25.2–46.2)	52.6 ± 15.6 (8.6–87.8)	23.9 ± 5.4 (17.0–40.7)	29.2 ± 4.9 (25.7–34.8)	53.4 ± 9.4 (34.2–72.5)	84.4 ± 12.1 (16.4–106.3)
Attack shape (unitless)	0.35 ± 0.04 (0.22–0.56)	0.40 ± 0.06 (0.02–0.59)	0.40 ± 0.05 (0.25–0.81)	0.37 ± 0.09 (0.27–0.42)	0.33 ± 0.05 (0.09–0.62)	0.34 ± 0.09 (0.01–0.70)
Decay shape (unitless)	0.33 ± 0.14 (0.10–0.60)	0.53 ± 0.15 (0.04–0.97)	0.59 ± 0.11 (0.34–0.91)	0.55 ± 0.15 (0.39–0.68)	0.64 ± 0.14 (0.12–0.96)	0.32 ± 0.26 (0.03–0.94)
Crest factor (unitless)	2.24 ± 0.03 (1.93–2.43)	2.29 ± 0.17 (1.68–3.62)	2.46 ± 0.16 (2.12–3.45)	2.47 ± 0.18 (2.33–2.68)	2.58 ± 0.24 (1.86–3.39)	2.21 ± 0.32 (1.70–3.21)
FREQUENCY TRAITS						
Frequency modulation	262.3 ± 63.2 (172–345)	351.0 ± 136.1 (0–646)	101.1 ± 64.7 (-50–258)	94.7 ± 40.4 (64–141)	108.4 ± 55.1 (-43–345)	97.9 ± 61.3 (-43–258)
Fundamental frequency	4211.6 ± 100.6 (4070–4328)	4461.6 ± 104.5 (4242–4931)	4812.0 ± 166.3 (4413–5470)	4617.0 ± 156.5 (4452–4763)	4188.9 ± 127.7 (3941–4414)	4447.8 ± 194.9 (4070–4974)
Dominant frequency	4223.0 ± 102.7 (4070–4328)	4527.7 ± 120.2 (4242–4888)	4810.4 ± 165.4 (4413–5467)	4612.8 ± 152.7 (4454–4758)	4189.6 ± 127.7 (4027–4414)	4461.6 ± 196.9 (4070–4974)
Attack frequency	4203.3 ± 110.8 (4070–4328)	4444.3 ± 104.4 (4242–4630)	4815.4 ± 164.9 (4417–5473)	4615.5 ± 159.4 (4447–4763)	4189.3 ± 132.8 (3941–4414)	4429.7 ± 196.7 (4070–4931)
Decay frequency	4465.6 ± 47.6 (4328–4587)	4795.3 ± 139.7 (4544–5189)	4916.5 ± 154.7 (4515–5551)	4710.2 ± 120.2 (4587–4828)	4297+8 ± 137.4 (4070–4630)	4527.6 ± 203.8 (4199–5103)
Bandwidth	228.6 ± 6.9 (196–306)	255.0 ± 33.1 (158–393)	258.4 ± 41.4 (210–494)	249.0 ± 35.2 (221–289)	234.0 ± 23.1 (159–360)	185.8 ± 21.2 (156–306)

The call duration varied from 28 to 91 ms in CCS Ma (n = 345 calls of 10 males) and the dominant frequency ranged from 4070 to 4888 Hz. In the CCS J, call duration from 21 to 45 ms (n = 813 calls of 17 males) and the dominant frequency from 4413 to 5467 Hz, whereas in CCS K call duration varied from 40 to 114 ms (n = 803 calls of 24 males) and the dominant frequency ranged from 4027 to 4974 Hz. All three CCSs had a frequency upsweep in their calls, but CCSs J and K also had calls without noticeable frequency modulation.

**Fig 6:**
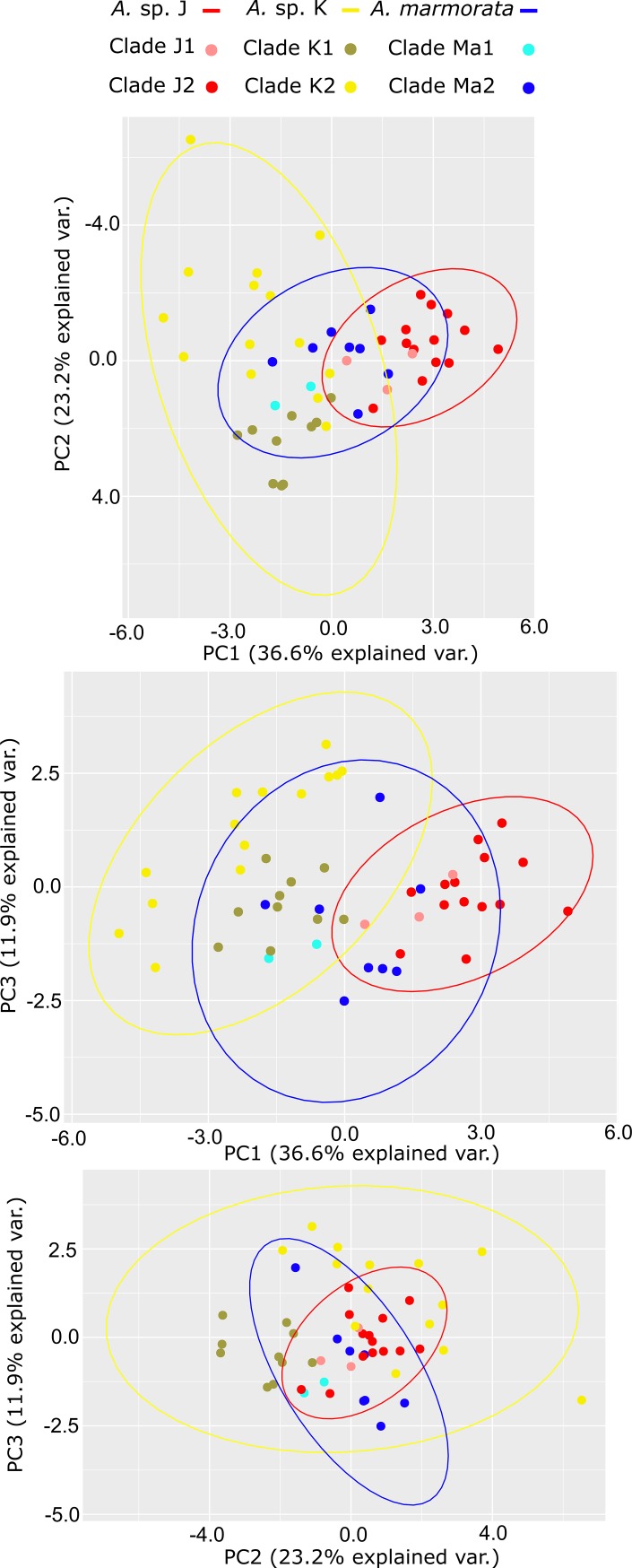
Principal Component Analysis (PCA) of call characters of CCS MA, CCS J and CCS K.

The first three factors of the Principal Component Analysis (PCA) accounted for 71.7% of the total variance in the acoustic trait space (36.6%, 23.2%, and 11.9%, respectively). The parameters that contributed the most for the differentiation of the proposed groups (CCSs Ma, J and K) were call attack frequency, call frequency modulation, call fundamental frequency and call decay frequency. The parameters ‘call rate’ and ‘rise time’ contributed the least to the first factor (PC1, Table 5). The graph provided by the PCA did not allow us to distinguish the lineages as distinct acoustic groups from each other (Fig 6).

**Table 5:** Results of the PCA of call variables of CCS Ma, CCAs J and CCS K.

	PC1	PC2	PC3
Call rate	0.009768	-0.18339	0.149232
Call duration	-0.32782	0.079983	0.38707
Rise time	-0.00721	0.357624	-0.3493
Attack shape	0.241929	-0.0441	0.316224
Decay shape	0.226465	-0.34494	0.017321
Crest factor	0.179381	-0.38706	0.154748
Dominant frequency	-0.02536	0.070557	-0.35227
Call frequency modulation	0.354363	0.253523	0.189933
Fundamental frequency	0.350815	0.267823	0.171745
Attack duration	-0.11316	0.418583	0.178111
Decay duration	-0.22677	0.317018	-0.16446
Plateau duration	-0.31021	-0.00398	0.430529
Call attack frequency	0.362484	0.228637	0.201825
Call decay frequency	0.348989	0.257257	0.049235
Bandwidth	0.295726	-0.16175	-0.32357
Eigenvalue	5.494109	3.484117	1.785174
% Total variance	36.63	23.23	11.9
Cumulative %	36.63	59.86	71.76

### Taxonomic decision

Considering acoustic and morphological variation in the CCS Ma, J and K, and the lack of diagnosable characters between them, we conclude that these three lineages are conspecifics. In order to clarify the taxonomy of *A*. *marmorata*, we now redescribe it based on our results.

#### Nomenclatural acts

**Fig 7:**
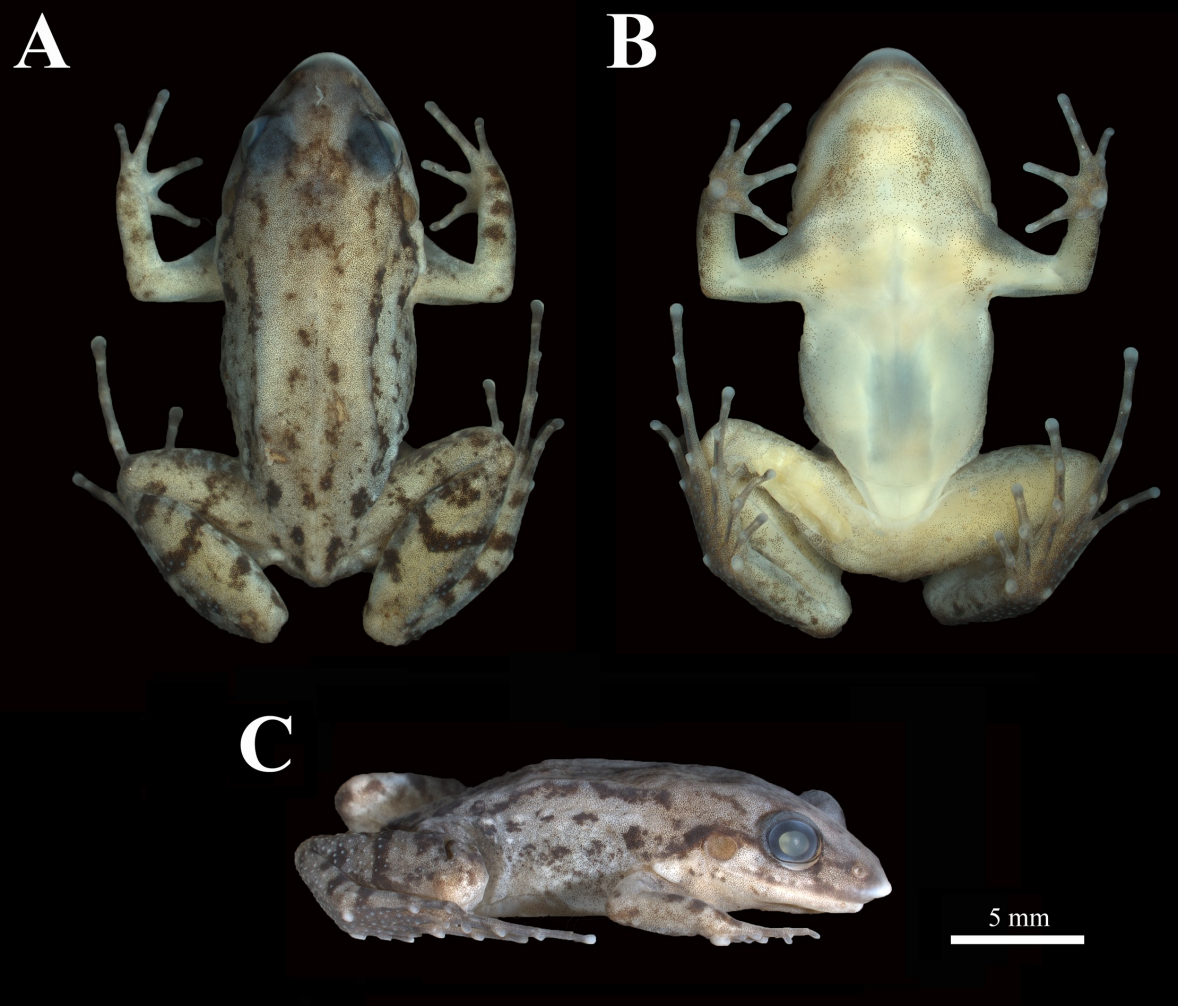
Topotype of *Adenomera marmorata*, CFBH 34403. (A) Dorsal, (B) Ventral, and (C) lateral views. Scale bar = 5 mm.

*Adenomera marmorata* Steindachner, 1867 (Figs 6 and 7)

Redescription

*Leptodactylus trivittatus* Lutz, 1926

*Leptodactylus* (*Parvulus*) *trivittatus*–Lutz, 1930

*Leptodactylus* (*Adenomera*) *marmorata*–Parker, 1932

*Leptodactylus marmoratus*–Parker, 1935

*Leptodactylus marmoratus marmoratus*–Rivero, 1961

*Adenomera marmorata–*Heyer, 1974

*Leptodactylus* (*Lithodytes*) *marmoratus*–Frost, Grant, Faivovich, Bain, Haas, Haddad, de Sá, Channing, Wilkinson, Donellan, Raxworthy, Campbell, Blotto, Moler, Drewes, Nussbaum, Lynch, Green, and Wheeler, 2006

*Adenomera marmorata*–Pyron and Wiens, 2011

*Adenomera marmorata*–Fouquet, Cassini, Haddad, Pech, and Rodrigues, 2014

*Adenomera* sp. J–Fouquet, Cassini, Haddad, Pech, and Rodrigues, 2014

*Adenomera* sp. K–Fouquet, Cassini, Haddad, Pech, and Rodrigues, 2014

**Fig 8:**
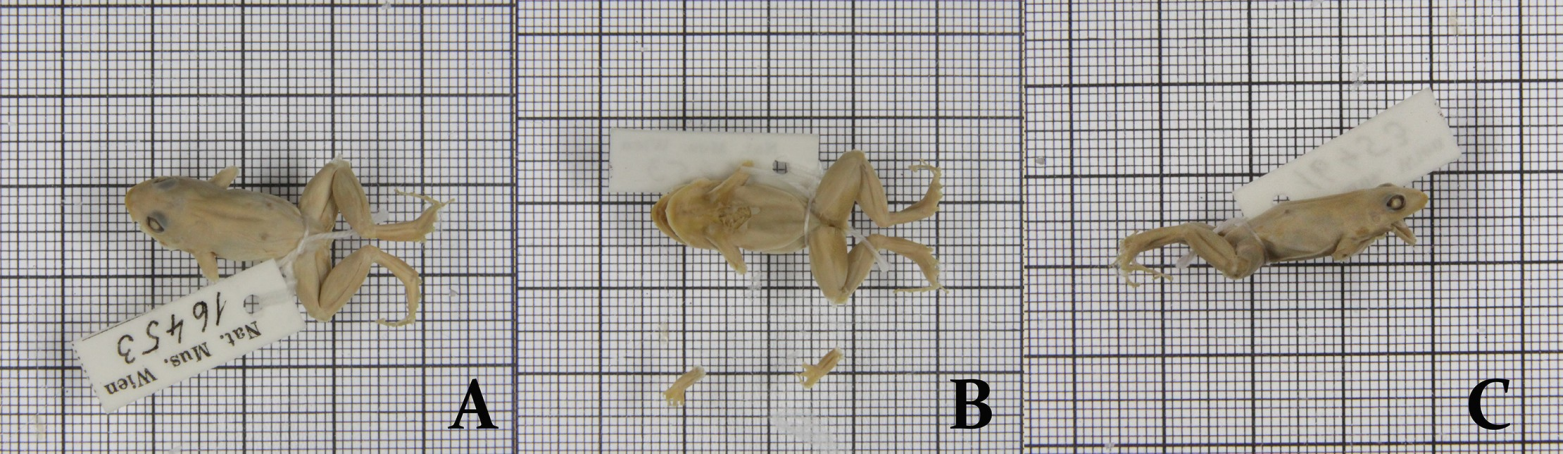
Holotype of *Adenomera marmorata*, NHMW 16453. Photographs by Dr. Heinz Grillitsch.

**Holotype.** NHMW 16453, adult male, Brazil (Fig 8).

#### Redescription of the holotype

The specimen is completely faded and with both forearms detached from the body, although some traits are still present. (Fig 8). Head longer than wide; HW 34% SVL, HL 39% SVL. Snout rounded in dorsal and ventral views; canthus rostralis indistinct. Loreal region slightly concave. Nostril not protruding, dorsolaterally oriented, rounded. Eye protruding, eye diameter larger than eye to nostril distance. Tympanum distinct, rounded, less than half the eye diameter. Supratympanic fold no apparent. Jaw glands absent. Vocal sac single, internal. Vomerine teeth could not be observed. Choanae moderate, rounded. Tongue elongate; lateral and posterior margins free. Vocal slits elongate, parallel to jaw, extending from posterior portion to the first third of the mouth. Arms poorly preserved, detached from the body. Toes free; toe tips II, III, and IV expanded (toe tips I and V unexpanded). Inner and outer metatarsal tubercles distinct and slightly protruding, ovoid, approximately the same size. Due to the degraded condition of the feet, toe formula could not be observed. Subarticular tubercles rounded and well-developed. Posterior members small and robust; thigh length slightly smaller than shank length (THL 40% SVL and SHL 44% SVL). Metatarsal fold absent. Tarsal fold present, poorly developed, extending from the base of outer metatarsal tubercle to the tibiotarsal junction. Ventral surface of tarsus with few tubercles. Ventral surface of feet with innumerous small and distinct tubercles. Dorsum smooth; anal glands present only on left side of the body, rounded.

#### Measurements of the Holotype

SVL 22.2 mm; HL 8.7 mm; HW 7.5 mm; ED 2.1 mm; TD 1.0 mm; END 1.3 mm; IND 1.4 mm; THL 8.8 mm; SHL 9.7 mm; TAL 5.3 mm. Measures of anterior members and feet could not be taken due to specimen condition.

#### Diagnosis

*Adenomera marmorata* is distinguished from congeners by the following combination of characters: (1) endotrophic tadpole; (2) toe tips II, III, and IV expanded; (3) side of the body with dark blotches; (4) antebrachial tubercle absent; (5) nearly solid dark-colored stripe along the underside of forearm absent; (6) dark-colored spots longitudinally arranged on dorsum absent; (7) nonpulsed advertisement call; (8) call duration varying from 21–114 ms; (9) dominant frequency ranging from 4027–5467 Hz; (10) call dominant frequency coinciding with the fundamental harmonic; (11) single-note advertisement call.

#### Comparisons with other species

*Adenomera marmorata* differs from *A*. *diptyx*, *A*. *saci*, and *A*. *thomei* by having endotrophic tadpoles (exotrophic in these species) [14, 22, 10]) The expanded toe tips (toes II–IV) of *Adenomera marmorata* distinguishes it from congeners with unexpanded toe tips (*A*. *bokermanni* [18], *A*. *coca*, *A*. *cotuba*, *A*. *diptyx*, *A*. *hylaedactyla*, *A*. *juikitam*, *A*. *kweti*, *A*. *martinezi*, *A*. *phonotriccus*, *A*. *saci*, and *A*. *thomei* [8–10, 14, 15, 61]).

*Adenomera marmorata* differs from *A*. *lutzi* and *A*. *phonotriccus* by the absence of black blotches on a yellow, orange, or red background on the posterior surface of the thigh and inguinal region (present in *A*. *lutzi*) and absence of antebrachial tubercle (present in *A*. *lutzi* and *A*. *phonotriccus*) [61, 62]; from *A*. *simonstuarti* by the absence of a nearly solid dark-colored stripe along the underside of forearm (present in *A*. *simonstuarti*) [9]; from *A*. *martinezi* and *A*. *saci* by the absence of 4 to 6 symmetrically arranged rows of longitudinal dark-colored spots on dorsum (rows present in *A*. *martinezi* and *A*. *saci*) [10]; from *A*. *araucaria* and *A*. *nana* by the presence of dark blotches on the side of the body (dark blotches absent or only small dark spots present in *A*. *araucaria* and *A*. *nana*).

The advertisement call of *Adenomera marmorata* distinguishes this species from all congeners, except *A*. *ajurauna* [28], by having its dominant frequency always coinciding with the fundamental harmonic (see Table 5). The calls of *Adenomera martinezi* [63] and *A*. *saci* [10] have their dominant frequencies coinciding with either the first or the second harmonics [14]. The nonpulsed advertisement call of *Adenomera marmorata* differs from those of pulsed-call species (*A*. *andreae*, *A*. *araucaria*, *A*. *chicomendesi*, *A*. *coca*, *A*. *cotuba*, *A*. *diptyx*, *A*. *heyeri*, *A*. *hylaedactyla*, *A*. *juikitam*, *A*. *martinezi*, *A*. *phonotriccus*, *A*. *simonstuarti*, and *A*. *thomei*) [8–10, 11, 12,14, 64, 65, 66, 67]; and from *A*. *ajurauna*, *A*. *heyeri*, and *A*. *juikitam* by a shorter advertisement call (21–114 ms in *A*. *marmorata*; 130–190 ms in *A*. *ajurauna* [28]; 137–185 ms in *A*. *heyeri* [64]; and 148–202 ms in *A*. *juikitam* [10, 14]. From the multi-note call *A*. *cotuba* [14] *A*. *marmorata* is distinguished by having a single-note call.

#### Color and Morphological variation

In life, general coloration of body grey; hind limbs with dark brown transversal stripes, posterior surface of the thigh with irregularly distributed melanophores on cream background. Heels with or without orangeade or reddish blotch. Venter cream, gular region with a few melanophore spots concentrated laterally. Lateral surface of the body with dark brown or black scattered blotches. Dorsum with marbled blotches irregularly scattered, without a clear pattern. Some specimens have two dorsolateral stripes, cream, orange, or red, extending from the posterior portion of the eye to the inguinal region. Dorsal surface of head with a bar or an inverted triangle homogeneously dark brown. Dark brown stripe contouring the tympanum from posterior portion of the eye to the arm-trunk junction. Forearms with dark brown transversal stripes or irregular blotches; hand with dorsal surface marbled.

In 70% alcohol preserved specimens, general gray coloration become yellowish or cream over time, evidencing the dark brown blotches and stripes but, at some point, they fade. Heel blotches and dorsolateral stripes become cream or white.

Tibial tubercles present or absent in some populations (Fig 4). Specimens from Alcatrazes Island are much larger than specimens from other populations. Snout subelliptical, subovoid, or rounded; loreal region vertical or oblique. Supernumerary tubercles of palmar region distinct or not.

#### Tadpole

The tadpole was described by Heyer *et al*. [52] and it is endotrophic, remaining in foam nests throughout the whole development.

#### Advertisement call

The advertisement call of *A*. *marmorata* is composed of single, nonpulsed notes having or not a frequency upsweep. Call duration varies from 21 to 114 ms, the dominant frequency coincides with the first harmonic and ranges from 4027 to 5467 Hz (Tables 4 and 6).

**Table 6:** Comparative advertisement call data for the Atlantic Forest species of *Adenomera*: call duration, pulses (presence/absence), and frequency peaks at the first two harmonics (H1 and H2, respectively). ^1^Based on the call of *Adenomera* sp. 2 from Joinville, Santa Catarina. Call data for *A*. *marmorata* comprise both clades of the nominal species (Ma1/Ma2) and the clades of the genetic lineages subsumed under the species (sp. J1/J2; sp. K1/K2; [7]). The dominant frequency of the calls of *A*. *ajurauna* and *A*. *marmorata* coincide with the first harmonic (H1); the other species have the dominant frequency of their calls coinciding with the second harmonic (H2).

Species	Call duration (ms)	Pulses/call	H1 frequency (kHz)	H2 frequency (kHz)	Reference
*A*. *ajurauna*	130–190	Nonpulsed	3.72–5.43	—	[28]
*A*. *araucaria*	102–277	4–18	2.10–2.48	4.11–4.93	[15]
*A*. *bokermanni*^1^	99–152	Nonpulsed	1.79–1.83	3.40–3.57	[13]
*A*. *engelsi*	96–163	Nonpulsed	~2.00	3.46–4.29	[16]
*A*. *kweti*	60–84	Nonpulsed	2.36–2.71	4.76–5.41	[15]
***A*. *marmorata***	**21–114**	**Nonpulsed**	**4.03–5.06**	**—**	**Present study**
*A*. *nana*	67–122	Nonpulsed	2.30–2.70	4.62–5.44	[13]
*A*. *thomei*	120–210	10–21	2.15–2.81	4.57–5.56	[22]

#### Natural history

*Adenomera marmorata* occurs mainly along forest edges and is highly abundant in most of its geographical distribution range. Males call on leaf-litter, exposed or not, near the constructed chamber for oviposition. The call activity may occur during all day in rainy days, but mostly from dusk into the first hours of night. On two occasions we observed males calling at dawn from inside collecting bags.

#### Geographic distribution and type-locality

*Adenomera marmorata* is distributed in Southeastern Brazil, over the “Serra da Mantiqueira” and northern part of the “Serra do Mar” mountain ranges of the states of Minas Gerais, Rio de Janeiro, and São Paulo, as well as in north-central coastal São Paulo, southern coastal Rio de Janeiro (see map on Fig 1); ranging in elevation from sea level up to more than 1200 m.

**Fig 9:**
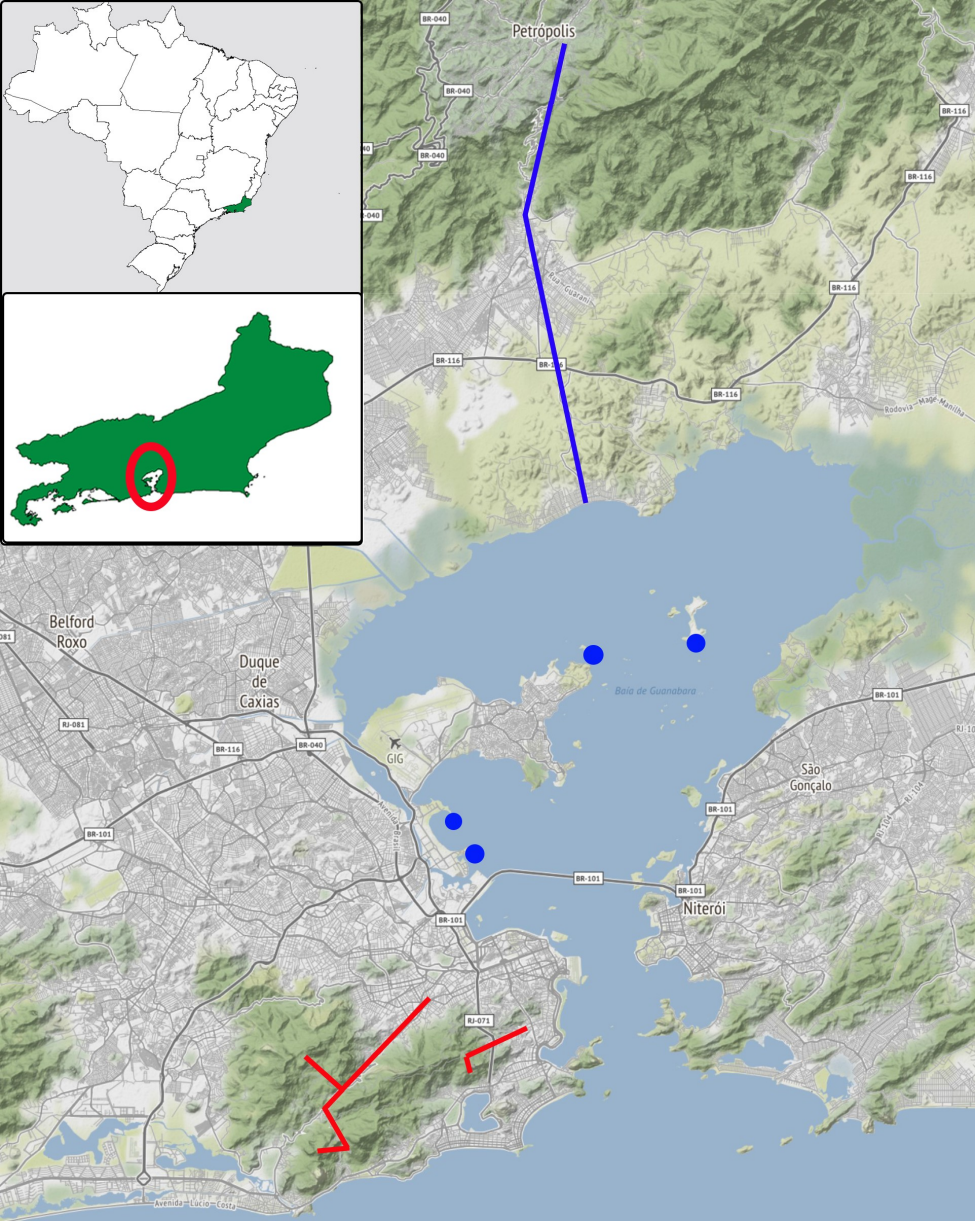
Traversed area by the “Novara Reise” [Novara Expedition] crew (August, 1857). In red, route taken for the city of Rio de Janeiro forested areas recognition, through the Tijuca Massif (type locality of *Adenomera marmorata*). In blue, route taken via rail and road transports to the municipality of Petrópolis. Blue dots indicate islands visited during an excursion through Guanabara Bay (image modified from INDE–Infraestrutura Nacional de Dados Espaciais, available at: https://visualizador.inde.gov.br/).

Steindachner [17] set the type-locality as “Brasilien” [Brazil]. The specimen was collected during the “Novara Reise” [17] which, in Brazil, only visited the municipality of Rio de Janeiro and its vicinity [19, 20]. During its brief stay in Brazil (August 5–31, 1857), the expedition crew went through several localities in the municipality of Rio de Janeiro [20] in the company of the Brazilian naturalist Dom Antonio Ildefonso Gomes, including the roads connecting the “Morro do Corcovado” (Corcovado hill) to, presumably, the “Pedra Bonita” (Beautiful Stone), currently in the Tijuca National Park, Tijuca Massif. The expedition members also went to the municipality of Petrópolis, going initially by train to the Fragoso station, municipality of Magé, and then finally to Petrópolis by rail and road transports through “Serra da Estrela” ([19], Fig 9). Since the goal of the travel to Petrópolis was to know and describe the German occupation at the locality and it was made by train and road transport [19], we consider unlikely that the holotype was collected at these locations (localities from Magé to Petrópolis municipalities). Because the objective of the expedition in the Tijuca region, at Rio de Janeiro city, was to describe the natural aspects of the place and the itinerary was surveyed by foot [19], we consider very likely that the holotype was collected somewhere on the road and we restrict the type-locality of *Adenomera marmorata* to the Tijuca Massif, municipality of Rio de Janeiro, Brazil.

#### Remarks

There are probably three species of *Adenomera* in the state of Rio de Janeiro. The first is the one we assigned to *A*. *marmorata*. Almeida & Angulo [22] reported a species of *Adenomera* from the municipality of Teresópolis, whose call duration (minimum call duration 247 ms) is markedly different from *A*. *marmorata* (maximum call duration 146 ms). These authors argued that due to differences in advertisement calls the specimen from Teresópolis municipality should not be the same species as the specimens from Tijuca Massif, Rio de Janeiro. We visited many localities also visited during the “Novara Reise” expedition and all analyzed specimens belongs to clade Ma2 (including the Tijuca Massif, the type locality). Fouquet *et al*. [7] suggested that the population of the CCS *Adenomera* sp. K (= CCS K) from the municipality of Itatiaia (Rio de Janeiro State) could either be conspecific with that population from Teresópolis [22] or correspond to *Leptodactylus trivittatus* (in this case would be under a new combination, *Adenomera trivittata*). Nevertheless, to date, none of the *Adenomera* species distributed in the Atlantic Forest has been reported to emit such long-lasting advertisement calls as reported by Almeida and Angulo [22] (Table 6). We collected and recorded two CCS from Itatiaia: *Adenomera marmorata* (formerly CCS K, Figs 1 and 2) and *A*. *thomei*. Heyer [18], when designating the lectotype of *Leptodactylus trivittatus* (see introduction), selected the specimen because it was the only one with the striped pattern mentioned by Lutz [23]. Nevertheless, no specimen of *A*. *thomei* (n = 75), among the specimens examined by us, had striped pattern, so we consider *A*. *thomei* and *L*. *trivittatus* as distinct species, maintaining Cochran’s [26] and Heyer’s [18] decision that *Leptodactylus trivittatus* is a junior synonym of *Adenomera marmorata*.

## Discussion

We used an integrative approach to test the hypothesis that the CCS Ma, CCS J and CCS K might correspond to different species. We used molecular phylogeny and raised considerably previous sampling of morphology and call characters. Our phenotypic data did not support the three lineages as three different species. Despite the genetic distance and reciprocal monophyly, these candidate species are not acoustically or morphologically diagnosable from each other.

### Tree topology and genetic distance thresholds

We recovered the same topology recovered by Fouquet *et al*. [7] regarding CCSs Ma, J and K using an extended sampling. The sole difference is the higher support values of the clade “CCS Ma + CCS J” (0.94 in [7] vs. 1.00 our results). The uncorrected *p*-distances among the three CCSs were high for both partial 16S and COI (Table 3). Fouquet *et al*. [68] suggested a mean distance of 3% on 16S to identify Neotropical anuran species and Vences *et al*. [69] found 10–14% of genetic divergence in mantellid frog species for COI. Even though the genetic divergence among the three lineages of *A*. *marmorata* is higher than the genetic distances between *A*. *bokermanni*, *A*. *engelsi*, and *A*. *nana*, it is known that genetic limits within a given genus may be very different (*e*.*g*. [69–71]). Fouquet *et al*. [7] tried to address this problem by taking as threshold the genetic distance between the two nominal species recovered as sister taxa in their study (*A*. *engelsi* and *A*. *nana*) as the first step to flag lineages as candidate species. The authors used additional lines of evidences, such as allele sharing, morphology, and bioacoustics to classify lineages as confirmed or unconfirmed candidate species. However, their analysis of acoustic and morphological divergence was superficial and their classification as CCS only tentative.

### Variation in a widespread species in the Atlantic Forest

*Adenomera marmorata* (as defined here) is one of the most abundant species found throughout forest edges of the Atlantic Forest in south-eastern Brazil. Although there are many studies assessing the genetic variation in widespread species (*e*.*g*. [72–74]), a few studies have described its phenotypic variation along all its geographical distribution (*e*.*g*. [75–77]). Furthermore, many species descriptions are based on few specimens from the same population (often because it is the only available material), which can lead to precipitated taxonomic conclusions. Our results show the robustness of delimiting species based on phenotypic and molecular datasets combined. We analyzed more phenotypic data from more localities than previously known for the *A*. *marmorata* clade (sensu Fouquet *et al*. [7]). The secondary lines of reproductive isolation evidences overlap among lineages, even those formerly used to distinguish one lineage from another (and thus, confirming the status of CCS of each lineage; Fouquet *et al*. [7]). Additionally, our PCA analysis was not able to distinguish any acoustic group.

The specimens of *A*. *marmorata* from the clade corresponding to CCS J, despite overlapping with the other two clades, showed a tendency to have a smaller SVL, shorter call length, and a higher dominant frequency. On the other hand, specimens of *A*. *marmorata*from Alcatrazes Island (CCS J) are much larger than specimens from other populations (male minimum SVL 25.6 mm, n = 1, female minimum SVL 25.7 mm, n = 4; other mainland populations male maximum SVL 22.4, n = 37, female maximum SVL = 23.4 mm, n = 24). Vertebrate gigantism on islands has massively been reported in the literature ([78–80]). Nevertheless, Rebouças *et al*. [80] found opposite results, (i.e. dwarfism) analyzing populations of *A*. *marmorata* from Ilha Grande Island. Variation in size of isolated insular communities may be related to genetic drift or to their local ecological processes, such as predation pressure, competition, and availability of resources [80–82].

Populations from CCSs J, K, and Ma were never found in syntopy. Nevertheless, the north coast of São Paulo deserves special attention. The northernmost distribution of CCSs J and Ma (clade Ma1) is on Ilha das Couves Island and Picinguaba, respectively—both localities within the municipal limits of Ubatuba, state of São Paulo. On the other hand, we identified only CCS K in the urban vicinities of Ubatuba. We should expect that, if these lineages represent indeed, distinct species, they should display any reproductive isolation mechanism in this ‘contact zone’. It is important to notice that Fouquet *et al*. [7] found spatial overlap among CCS Ma, J and *K* but no allele sharing between them. Nevertheless, we found that populations of CCSs Ma, J and K from Ubatuba and its vicinities share a haplotype (h16). Additionally, all morphological and acoustic parameters overlapped among these populations and no evidence of such distinctiveness could be found.

Campos *et al*. [83] studied the karyotype, among other leptodactylid species, from the three clades of *A*. *marmorata*, and their results corroborate the hypothesis that they are the same species. We analyzed two specimens from that study: CFBH 11512, from the municipality of Santa Branca, state of São Paulo (corresponding to CCS Ma) and CFBH 17137, from Alcatrazes Island, state of São Paulo (corresponding to CCS J). No cytogenetic difference was found between the two samples [83]. However, their samples from the municipalities of Salesópolis and São Luís do Paraitinga, state of São Paulo (which correspond J and CCS Ks in our study, respectively) showed the chromosome pair 12 metacentric, differing from the specimens from Santa Branca and Alcatrazes Island, which had the same chromosome pair telocentric. Campos *et al*. [83] suggested that this difference was due to intraspecific geographic variation and the data presented herein corroborate that interpretation.

It is important to point out that we assessed the taxonomic status of *A*. *marmorata* using molecular, acoustic, and morphological data, but there are still other character sets to be assessed (*e*.*g*. ecology, behavior, and tadpole morphology). As noted by de Queiroz [27], closely related species might be in “gray zones” (*i*.*e*., time in species evolution where alternative species concepts and character sets come into conflict, making it difficult for us to recognize species) and additional characters put into analysis may shed new light on the taxonomy of *A*. *marmorata* and lead to a more precise interpretation.

*Adenomera marmorata* is a widespread species showing rich phylogenetic structure and remarkable acoustical and morphological variation. These aspects make this species an interesting model to studies on diversification processes in the Atlantic Forest.

## Supporting information

S1 Fig Bayesian inference and genetic samples. 50% majority rule consensus tree from Bayesian inference analysis of concatenated nuclear (POMC and RAG) and mitochondrial (COI, CYTB, and 16S).
(TRE)

S2 Fig Phylogenetic analyses of maximum likelihood. (TRE)

S1 Table Sample information of *Adenomera* species. Corresponding clade, voucher or field number, locality information (country or State when in Brazil; locality name, coordinates), and GenBank accession number.
(DOCX)

S2 Table Information associated with analyzed sound files of the six lineages within Adenomera marmorata (see Methods). Calls were recorded from the Brazilian Atlantic Forest.
(DOCX)

S3 Table Acoustic terminology and definitions for the automated analysis of acoustic traits. Temporal traits were obtained from waveforms; spectral traits from spectrograms and amplitude spectra. RMS = root mean square.
(DOCX)
