# Supplementary material for: One step beyond a broad molecular phylogenetic analysis: Species delimitation of Adenomera marmorata Steindachner, 1867 (Anura: Leptodactylidae)
Source: PLoS One. 2020 Feb 21;15(2):e0229324. doi: 10.1371/journal.pone.0229324 (PMC7034910; doi:10.1371/journal.pone.0229324)
Supplement: S1 Table — Corresponding clade, voucher or field number, locality information (country or State when in Brazil; locality name, coordinates), and GenBank accession number. (DOCX) [file pone.0229324.s003.docx]

**SUPPORTING INFORMATION**

**One step beyond a broad molecular phylogenetic analysis: Species delimitation of *Adenomera marmorata* Steindachner, 1867 (Anura: Leptodactylidae)**

Carla S. Cassini, Pedro P. G. Taucce, Thiago R. de Carvalho; Antoine Fouquet, Célio F. B. Haddad and Paulo C. A. Garcia

*Plos One*

**S1 Table. Sample information of *Adenomera* species**. Corresponding clade, voucher or field number, locality information (country or State when in Brazil; locality name, coordinates) and GenBank accession number. Sequences generated in this study are in bold.

| **Terminal** | **Species** | **Clade** | **Voucher** | **State** | **Locality** | **Latitude** | **Longitude** | **16S** | **Cyt b** | **COI** | **RAG1** | **POMC** |
| --- | --- | --- | --- | --- | --- | --- | --- | --- | --- | --- | --- | --- |
| A_A_MNRJ47474 | *Adenomera* sp.A |  | MNRJ47474 | RJ | Macaé, Reserva Biológica União | – 22.49130 | – 42.001133 | KF675185 | KF675112 | KF674803 | KF674491 | KF674171 |
| A_ajurauna_BB459 | *A. ajurauna* |  | BB459 | SP | Mogi das Cruzes, Parque das Neblinas | – 23.735200 | – 46.180252 | **MT035972** | **MT041260** | **MT036115** | **MT041497** | **MT041390** |
| A_ajurauna_CTMZ01602 | *A. ajurauna* |  |  | SP | Embu | – 23.65055 | – 46.850833 | –– | KF675081 | KF674772 | KF674459 | KF674140 |
| A_ajurauna_CTMZ01932 | *A. ajurauna* |  |  | SP | Jaceguava | – 23.75916 | – 46.778055 | –– | KF675082 | KF674773 | KF674460 | KF674141 |
| A_ajurauna_CTMZ02393 | *A. ajurauna* |  |  | SP | P.E. Carlos Botelho, São Miguel Arcanjo | – 24.05075 | – 47.992802 | –– | KF675079 | KF674770 | KF674457 | KF674138 |
| A_ajurauna_H182 | *A. ajurauna* |  |  | SP | Juquitiba | – 23.92988 | – 47.066832 | KF675179 | KF675080 | KF674771 | KF674458 | KF674139 |
| A_ajurauna_H33 | *A. ajurauna* |  |  | SP | Piedade | – 23.6875 | – 47.4375 | –– | KF675083 | KF674774 | KF674461 | KF674142 |
| A_ajurauna_MZUSPfield1548 | *A. ajurauna* |  | MZUSPfield1548 | SP | Paranabiacaba | – 23.777710 | – 46.295539 | **MT035973** | **MT041261** | **MT036116** | **MT041498** | **MT041391** |
| A_andreae_317AF | *A. andreae* |  |  | FG | Parare Left | 4.042.520 | – 52.67879 | KC520683 | JQ321766 | KC520689 | KF674220 | KC604061 |
| A_andreae_MHNC10031 | *A. andreae* |  |  | Peru | Mashira JX, Dist. Rio Tambo, Prov. Satipo, Dpto. Junin. | – 11.21322 | – 73.452054 | –– | KF674843 | KF674531 | KF674211 | KF673899 |
| A_andreae_MTR20955 | *A. andreae* |  |  | RR | Tepequem | 3.728.255 | – 61.698904 | –– | KF674832 | KF674520 | KF674200 | KF673888 |
| A_araucaria_EMLG0609 | *A. araucaria* |  |  | SC | Vidal Ramos | – 27.39849 | – 49.378409 | –– | KF675063 | KF674754 | KF674439 | KF674122 |
| A_araucaria_MCP10769 | *A. araucaria* |  | MCP10769 | RS | São Francisco de Paula | – 29.34894 | – 50.182214 | KC603944 | KC603969 | KC603997 | KF674440 | KC604065 |
| A_araucaria_MCP11241 | *A. araucaria* |  | MCP11241 | RS | Floresta Nacional São Francisco de Paula | – 29.46214 | – 50.568881 | –– | KF675064 | KF674755 | KF674441 | KF674123 |
| A_B_ESTR307 | *A.* sp.B |  |  | MA | Carolina | – 7.22941 | – 47.26147 | KF675169 | KF675007 | KF674698 | KF674381 | KF674066 |
| A_bokermanni_K1730 | *A. bokermanni* |  |  | PR | Paranaguá | – 25.52261 | – 48.556252 | –– | KF675115 | KF674806 | KF674494 | KF674174 |
| A_bokermanni_MTR18508 | *A. bokermanni* |  |  | PR | Morro Grande Guaratuba | – 25.97130 | – 48.71403 | KF675187 | KF675116 | KF674807 | KF674495 | KF674175 |
| A_C_MNCN34687 | *A.* sp.C |  |  | BOL | Iturralde, La paz | – 12.68 | – 68.711666 | –– | KF674878 | KF674568 | KF674249 | KF673936 |
| A_C_USNM268935 | *A.* sp.C |  |  | FG | Nouragues2 | 40.916.666 | – 52.7 | –– | KF675009 | KF674700 | KF674383 | KF674068 |
| A_D_ZSM751 | *A.* sp.D |  |  | Peru | Huanuco, Est Bio Panguana | – 9.603932 | – 74.935799 | KF675165 | KF674881 | KF674571 | KF674252 | KF673939 |
| A_diptyx_IIBPH905 | *A. diptyx* |  |  | PAR | Concepción, Cerrados del Tagatiyá | – 22.72 | – 57.37 | KC603938 | KC603966 | KC603994 | KF674278 | KC604062 |
| A_diptyx_RGA5254 | *A. diptyx* |  |  | MT | UHE Guaporé | – 15.33333 | – 58.85 | –– | KF674910 | KF674601 | KF674282 | KF673969 |
| A_E_AA9972 | *A.* sp.E |  |  | Peru | Madre de dios, Tambopata | – 14.22361 | – 69.176667 | KF675167 | JQ321831 | KF674586 | KF674267 | KF673954 |
| A_E_ZUFG108 | *A.* sp.E |  |  | AC | Rio Branco (UFAM) | – 9.944835 | – 67.800339 | –– | KF674901 | KF674592 | KF674273 | KF673960 |
| A_engelsi_MNRJ72224 | *A. engelsi* |  | MNRJ72224 | SC | São Bonifácio | – 27.90341 | – 48.933907 | –– | KF675073 | KF674764 | KF674451 | KF674132 |
| A_engelsi_MTR18497 | *A. engelsi* |  |  | SC | Praia dos naufragados, Florianopolis | – 27.82361 | – 48.56168 | KC603940 | KC603970 | KC603998 | KF674450 | KC604065 |
| A_engelsi_TRPD1 | *A. engelsi* |  |  | SC | Bombinhas | – 27.19158 | – 48.56904 | –– | KF675075 | KF674766 | KF674453 | KF674134 |
| A_engelsi_ZUFG093 | *A. engelsi* |  |  | SC | Itapema | – 27.08934 | – 48.612986 | –– | KF675074 | KF674765 | KF674452 | KF674133 |
| A_F_AF301 | *A.* sp.F |  |  | MT | Vila Rica | – 9.924166 | – 51.238333 | –– | KF675016 | KF674707 | KF674391 | KF674075 |
| A_F_MTR11092 | *A.* sp.F |  |  | PA | Floresta Nacional do Tapajós | – 3.5 | – 55.066666 | KF675170 | KF675023 | KF674714 | KF674398 | KF674082 |
| A_G_MTR12711 | *A.* sp.G |  |  | AM | Igarapé Açu, Rio Abacaxis | – 4.344166 | – 58.635 | –– | KF675024 | KF674715 | KF674399 | KF674083 |
| A_G_MTR12832 | *A.* sp.G |  |  | AM | São Sebastião, Rio Abacaxis | – 4.308889 | – 58.636389 | KF675171 | KF675026 | KF674717 | KF674401 | KF674085 |
| A_H_AF970 | *A.* sp.H |  |  | TO | Palmas | – 10.15592 | – 48.332405 | KF675172 | KF675031 | KF674722 | KF674406 | KF674090 |
| A_H_CHUNB46027 | *A.* sp.H |  |  | TO | Caseara | – 9.259154 | – 49.952202 | –– | KF675030 | KF674721 | KF674405 | KF674089 |
| A_heyeri_127AF | *A. heyeri* |  |  | SUR | Brownsberg | 4.917.201 | – 55.220718 | KC603948 | KC603972 | KC604000 | KF674387 | KC604068 |
| A_heyeri_269AF | *A. heyeri* |  |  | FG | Nouragues2 | 40.916.666 | – 52.7 | –– | KF675009 | KF674700 | KF674383 | KF674068 |
| A_hylaedactyla_CTMZ06288 | *A. hylaedactyla* |  |  | PA | Jacareacanga | – 6.222222 | – 57.752777 | –– | KF674979 | KF674670 | KF674352 | KF674038 |
| A_hylaedactyla_MTR21603 | *A. hylaedactyla* |  |  | GO | Pirenópolis | – 15.84510 | – 48.9608 | –– | KF674991 | KF674682 | KF674364 | KF674050 |
| A_hylaedactyla_UFMT2 | *A. hylaedactyla* |  |  | MT | Cuiabá | – 15.59615 | – 56.038914 | –– | KF674987 | KF674678 | KF674360 | KF674046 |
| A_hylaedactyla_ZUFG101 | *A. hylaedactyla* |  |  | PA | Peixe-boi | – 1.193098 | – 47.302437 | KC603939 | KC603967 | KC603995 | KF674302 | KC604063 |
| A_I_CHUNB27648 | *A.* sp.I |  |  | TO | Mateiros | – 5.248811 | – 48.199482 | KC603950 | KC603973 | KC604001 | KF674417 | KC604069 |
| A_I_CHUNB46034 | *A.* sp.I |  |  | TO | Caseara | – 9.259154 | – 49.952202 | –– | KF675038 | KF674729 | KF674413 | KF674097 |
| A_J_AF1506 | *A. marmorata* | J |  | SP | Santos | – 23.93442 | – 46.335068 | –– | KF675057 | KF674748 | KF674433 | KF674116 |
| A_J_AF1562 | *A. marmorata* | J |  | SP | São Vicente | – 23.96305 | – 46.391944 | KF675175 | KF675056 | KF674747 | KF674432 | KF674115 |
| A_J_AF771 | *A. marmorata* | J |  | SP | Barra do Una | – 23.75 | – 45.75 | –– | KF675058 | KF674749 | KF674434 | KF674117 |
| A_J_BB292 | *A. marmorata* | J | BB292 | SP | Mogi das Cruzes, Parque das Neblinas | – 23.74204 | – 46.135903 | –– | **MT041262** | **MT036117** | **MT041499** | –– |
| A_J_BB296 | *A. marmorata* | J | BB296 | SP | Mogi das Cruzes, Parque das Neblinas | – 23.735200 | – 46.180252 | **MT035974** | **MT041263** | **MT036118** | **MT041500** | –– |
| A_J_BB311 | *A. marmorata* | J | BB311 | SP | Mogi das Cruzes, Parque das Neblinas | – 23.74204 | – 46.135903 | **MT035975** | **MT041264** | **MT036119** | **MT041501** | –– |
| A_J_BB319 | *A. marmorata* | J | BB319 | SP | Mogi das Cruzes, Parque das Neblinas | – 23.735200 | – 46.180252 | **MT035976** | **MT041265** | **MT036120** | **MT041502** | –– |
| A_J_CFBH11355 | *A. marmorata* | J | CFBH11355 | SP | Cubatão | – 23.841688 | – 46.396604 | –– | **MT041266** | **MT036121** | **MT041503** | **MT041392** |
| A_J_CFBH11542 | *A. marmorata* | J | CFBH11542 | SP | Cubatão | – 23.841688 | – 46.396604 | –– | **MT041267** | **MT036122** | **MT041504** | **MT041393** |
| A_J_CFBH12236 | *A. marmorata* | J | CFBH12236 | SP | Biritiba Mirim | – 23.556879 | – 46.0998082 | –– | **MT041268** | **MT036123** | **MT041505** | **MT041394** |
| A_J_CFBH12305 | *A. marmorata* | J | CFBH12305 | SP | Cubatão | – 23.841688 | – 46.396604 | –– | **MT041269** | **MT036124** | **MT041506** | **MT041395** |
| A_J_CFBH12884 | *A. marmorata* | J | CFBH12884 | SP | São Sebastião, Ilha das Couves | – 23.420331 | – 44.852791 | –– | **MT041270** | **MT036125** | **MT041507** | –– |
| A_J_CFBH15953 | *A. marmorata* | J | CFBH15953 | SP | Santos | – 23.948586 | – 46.352898 | –– | **MT041271** | **MT036126** | **MT041508** | –– |
| A_J_CFBH17137 | *A. marmorata* | J | CFBH17137 | SP | Ilha de Alcatrazes | – 24.100177 | – 45.690083 | –– | **MT041272** | **MT036127** | **MT041509** | **MT041396** |
| A_J_CFBH17138 | *A. marmorata* | J | CFBH17138 | SP | Ilha de Alcatrazes | – 24.100177 | – 45.690083 | –– | **MT041273** | **MT036128** | **MT041510** | **MT041397** |
| A_J_CFBH17139 | *A. marmorata* | J | CFBH17139 | SP | Ilha de Alcatrazes | – 24.100177 | – 45.690083 | **MT035977** | **MT041274** | **MT036129** | **MT041511** | **MT041398** |
| A_J_CFBH17140 | *A. marmorata* | J | CFBH17140 | SP | Ilha de Alcatrazes | – 24.100177 | – 45.690083 | –– | **MT041275** | **MT036130** | **MT041512** | **MT041399** |
| A_J_CFBH17141 | *A. marmorata* | J | CFBH17141 | SP | Ilha de Alcatrazes | – 24.100177 | – 45.690083 | **MT035978** | **MT041276** | **MT036131** | **MT041513** | **MT041400** |
| A_J_CFBH17142 | *A. marmorata* | J | CFBH17142 | SP | Ilha de Alcatrazes | – 24.100177 | – 45.690083 | –– | **MT041277** | **MT036132** | **MT041514** | **MT041401** |
| A_J_CFBH17143 | *A. marmorata* | J | CFBH17143 | SP | Ilha de Alcatrazes | – 24.100177 | – 45.690083 | **MT035979** | **MT041278** | **MT036133** | **MT041515** | **MT041402** |
| A_J_CFBH20218 | *A. marmorata* | J | CFBH20218 | SP | São Sebastião, Ilha das Couves | – 23.420331 | – 44.852791 | **MT035980** | **MT041279** | **MT036134** | **MT041516** | **MT041403** |
| A_J_CFBH20219 | *A. marmorata* | J | CFBH20219 | SP | São Sebastião, Ilha das Couves | – 23.420331 | – 44.852791 | –– | **MT041280** | **MT036135** | –– | **MT041404** |
| A_J_CFBH20222 | *A. marmorata* | J | CFBH20222 | SP | São Sebastião, Ilha das Couves | – 23.420331 | – 44.852791 | **MT035981** | **MT041281** | **MT036136** | **MT041517** | **MT041405** |
| A_J_CFBH23923 | *A. marmorata* | J | CFBH23923 | SP | Santos | – 23.948586 | – 46.352898 | –– | **MT041282** | **MT036137** | **MT041518** | **MT041406** |
| A_J_CFBH32816 | *A. marmorata* | J | CFBH32816 | SP | Itanhanhém | – 24.148446 | – 46.857202 | **MT035982** | **MT041283** | **MT036138** | **MT041519** | **MT041407** |
| A_J_CFBH32818 | *A. marmorata* | J | CFBH32818 | SP | Mogi das Cruzes, Parque das Neblinas | – 23.735200 | – 46.180252 | –– | **MT041284** | **MT036139** | **MT041520** | **MT041408** |
| A_J_CFBH32819 | *A. marmorata* | J | CFBH32819 | SP | Mogi das Cruzes, Parque das Neblinas | – 23.735200 | – 46.180252 | –– | **MT041285** | **MT036140** | **MT041521** | –– |
| A_J_CFBH9212 | *A. marmorata* | J | CFBH9212 | SP | Cubatão, COPEBÁS | – 23.841688 | – 46.396604 | –– | **MT041286** | **MT036141** | **MT041522** | **MT041409** |
| A_J_CFBHt14300 | *A. marmorata* | J | CFBHt14300 | SP | Mogi das Cruzes, Parque das Neblinas | – 23.74204 | – 46.135903 | –– | **MT041287** | **MT036142** | **MT041523** | –– |
| A_J_CFBHt14301 | *A. marmorata* | J | CFBHt14301 | SP | Paranapiacaba | – 23.777710 | – 46.295539 | **MT035983** | **MT041288** | **MT036143** | **MT041524** | –– |
| A_J_CFBHt14303 | *A. marmorata* | J | CFBHt14303 | SP | Mogi das Cruzes, Parque das Neblinas | – 23.735200 | – 46.180252 | –– | **MT041289** | **MT036144** | **MT041525** | –– |
| A_J_CFBHt14575 | *A. marmorata* | J | CFBHt14575 | SP | Mogi das Cruzes, Parque das Neblinas | – 23.735200 | – 46.180252 | –– | **MT041290** | **MT036145** | **MT041526** | –– |
| A_J_CFBHt3854 | *A. marmorata* | J | CFBHt3854 | SP | Itanhanhém | – 24.148446 | – 46.857202 | –– | **MT041291** | **MT036146** | –– | **MT041410** |
| A_J_CFBHt3908 | *A. marmorata* | J | CFBHt3908 | SP | São Bernado do Campo | – 23.731470 | – 46.611932 | –– | **MT041292** | **MT036147** | **MT041527** | **MT041411** |
| A_J_CFBHt5823 | *A. marmorata* | J | CFBHt5823 | SP | São Sebastião, Ilha das Couves | – 23.420331 | – 44.852791 | –– | **MT041293** | **MT036148** | –– | **MT041412** |
| A_J_CFBHt5853 | *A. marmorata* | J | CFBHt5853 | SP | Biritiba Mirim | – 23.556879 | – 46.0998082 | –– | **MT041294** | **MT036149** | –– | **MT041413** |
| A_J_CFBHt633 | *A. marmorata* | J | CFBHt633 | SP | Ilha de Alcatrazes | – 24.09865 | – 45.694127 | –– | **MT041295** | **MT036150** | **MT041528** | **MT041414** |
| A_J_CTMZ01611 | *A. marmorata* | J |  | SP | Santo André, Parque do Pedroso | – 23.72694 | – 46.505277 | –– | KF675059 | KF674750 | KF674435 | KF674118 |
| A_J_H566 | *A. marmorata* | J |  | SP | Bertioga | – 23.85444 | – 46.138611 | –– | KF675055 | KF674746 | KF674431 | KF674114 |
| A_J_Ialc2 | *A. marmorata* | J |  | SP | Ilha de Alcatrazes | – 24.09865 | – 45.694127 | –– | KF675061 | KF674752 | KF674437 | KF674120 |
| A_J_ITH63_AF1047 | *A. marmorata* | J |  | SP | São Bernado | – 23.72815 | – 46.518974 | –– | KF675054 | KF674745 | KF674430 | KF674113 |
| A_J_MZUSP0257 | *A. marmorata* | J | MZUSP0257 | SP | Ribeirão Pires | –23.72339 | –46.379843 | –– | KF675060 | KF674751 | KF674436 | KF674119 |
| A_J_MZUSPfield1547 | *A. marmorata* | J | MZUSPfield1547 | SP | Paranapiacaba | – 23.777710 | – 46.295539 | –– | **MT041296** | **MT036151** | **MT041529** | **MT041415** |
| A_J_MZUSPfield1549 | *A. marmorata* | J | MZUSPfield1549 | SP | Paranapiacaba | – 23.777710 | – 46.295539 | **MT035984** | **MT041297** | **MT036152** | **MT041530** | **MT041416** |
| A_J_MZUSPfield1550 | *A. marmorata* | J | MZUSPfield1550 | SP | Paranapiacaba | – 23.777710 | – 46.295539 | **MT035985** | **MT041298** | **MT036153** | **MT041531** | **MT041417** |
| A_J_MZUSPfield1568 | *A. marmorata* | J | MZUSPfield1568 | SP | Paranapiacaba | – 23.777710 | – 46.295539 | –– | **MT041299** | **MT036154** | **MT041532** | **MT041418** |
| A_J_UFMG2708 | *A. marmorata* | J | UFMG2708 | SP | Mogi das Cruzes, Parque das Neblinas | – 23.74204 | – 46.135903 | –– | **MT041300** | **MT036155** | **MT041533** | **MT041419** |
| A_J_UFMG2709 | *A. marmorata* | J | UFMG2709 | SP | Mogi das Cruzes, Parque das Neblinas | – 23.74204 | – 46.135903 | –– | **MT041301** | **MT036156** | **MT041534** | **MT041420** |
| A_J_UFMG9470 | *A. marmorata* | J | UFMG9470 | SP | Paranapiacaba | – 23.777710 | – 46.295539 | –– | **MT041302** | **MT036157** | **MT041535** | **MT041421** |
| A_J_UFMG9471 | *A. marmorata* | J | UFMG9471 | SP | Paranapiacaba | – 23.777710 | – 46.295539 | –– | **MT041303** | **MT036158** | **MT041536** | **MT041422** |
| A_J_USNM303076 | *A. marmorata* | J |  | SP | Salesópolis, E.B. de Boraceia | – 23.63305 | – 45.533055 | –– | KF675062 | KF674753 | KF674438 | KF674121 |
| A_K_CFBH19968 | *A. marmorata* | K | CFBH19968 | SP | Ubatuba | – 23.416232 | – 45.072105 | –– | **MT041304** | **MT036159** | **MT041537** | **MT041423** |
| A_K_CFBH19969 | *A. marmorata* | K | CFBH19969 | SP | Ubatuba, Instituto Agronômico | – 23.433992 | – 45.102659 | **MT035986** | **MT041305** | **MT036160** | **MT041538** | **MT041424** |
| A_K_CFBH35983 | *A. marmorata* | K | CFBH35983 | MG | Muriaé | – 21.123348 | – 42.375606 | **MT035987** | **MT041306** | **MT036161** | **MT041539** | **MT041425** |
| A_K_CFBH35995 | *A. marmorata* | K | CFBH35995 | SP | São Luís do Paraitinga | – 23.36753 | – 45.14284 | **MT035988** | **MT041307** | **MT036162** | **MT041540** | **MT041426** |
| A_K_CFBH35996 | *A. marmorata* | K | CFBH35996 | SP | São Luís do Paraitinga | – 23.36753 | – 45.14284 | –– | **MT041308** | **MT036163** | **MT041541** | **MT041427** |
| A_K_CFBH35998 | *A. marmorata* | K | CFBH35998 | SP | São Luís do Paraitinga | – 23.36753 | – 45.14284 | –– | **MT041309** | **MT036164** | **MT041542** | **MT041428** |
| A_K_CFBH36000 | *A. marmorata* | K | CFBH36000 | SP | Ubatuba, Praia Vermelha | – 23.417777 | – 45.03828 | –– | **MT041310** | **MT036165** | **MT041543** | **MT041429** |
| A_K_CFBH36001 | *A. marmorata* | K | CFBH36001 | SP | Ubatuba, Praia Vermelha | – 23.417777 | – 45.03828 | **MT035989** | **MT041311** | **MT036166** | **MT041544** | **MT041430** |
| A_K_CFBH36002 | *A. marmorata* | K | CFBH36002 | SP | Ubatuba, Praia Vermelha | – 23.417777 | – 45.03828 | –– | **MT041312** | **MT036167** | **MT041545** | **MT041431** |
| A_K_CFBH36005 | *A. marmorata* | K | CFBH36005 | SP | Ubatuba, Mirante do centro | – 23.43230 | – 45.068194 | –– | **MT041313** | **MT036168** | **MT041546** | **MT041432** |
| A_K_CFBH36006 | *A. marmorata* | K | CFBH36006 | SP | Ubatuba, Mirante do centro | – 23.43230 | – 45.068194 | –– | **MT041314** | **MT036169** | **MT041547** | **MT041433** |
| A_K_CFBHt14292 | *A. marmorata* | K | CFBHt14292 | RJ | Rio Claro, P.E. do Cunhambebe | – 22.821651 | – 44.003151 | –– | **MT041315** | **MT036170** | **MT041548** | **MT041434** |
| A_K_CFBHt15856 | *A. marmorata* | K | CFBHt15856 | RJ | Itatiaia, Pousada Chalés Terra Nova | – 22.4565 | – 44.585194 | –– | **MT041316** | **MT036171** | **MT041549** | **MT041435** |
| A_K_CFBHt8938 | *A. marmorata* | K | CFBHt8938 | SP | Ubatuba | – 23.416232 | – 45.072105 | –– | **MT041317** | **MT036172** | **MT041550** | **MT041436** |
| A_K_MNRJ74640 | *A. marmorata* | K | MNRJ74640 | RJ | Rio Claro, Lídice | – 22.832788 | – 44.188550 | KF675184 | KF675111 | KF674802 | KF674490 | KF674170 |
| A_K_MTR15574 | *A. marmorata* | K | MTR15574 | RJ | Penedo, Itatiaia | – 22.43333 | – 44.533333 | –– | KF675110 | KF674801 | –– | KF674169 |
| A_K_UFMG13284 | *A. marmorata* | K | UFMG13284 | RJ | Itatiaia | – 22.4565 | – 44.585194 | –– | **MT041318** | **MT036173** | **MT041551** | **MT041437** |
| A_K_UFMG13285 | *A. marmorata* | K | UFMG13285 | RJ | Itatiaia | – 22.4565 | – 44.585194 | –– | **MT041319** | **MT036174** | **MT041552** | **MT041438** |
| A_K_UFMG13287 | *A. marmorata* | K | UFMG13287 | RJ | Itatiaia | – 22.4565 | – 44.585194 | –– | **MT041320** | **MT036175** | **MT041553** | –– |
| A_K_UFMG13288 | *A. marmorata* | K | UFMG13288 | RJ | Itatiaia | – 22.4565 | – 44.585194 | –– | **MT041321** | **MT036176** | **MT041554** | –– |
| A_K_UFMG13289 | *A. marmorata* | K | UFMG13289 | RJ | Itatiaia | – 22.4565 | – 44.585194 | **MT035990** | **MT041322** | **MT036177** | **MT041555** | **MT041439** |
| A_K_UFMG13290 | *A. marmorata* | K | UFMG13290 | RJ | Itatiaia | – 22.4565 | – 44.585194 | –– | **MT041323** | **MT036178** | **MT041556** | **MT041440** |
| A_K_UFMG13291 | *A. marmorata* | K | UFMG13291 | RJ | Itatiaia | – 22.4565 | – 44.585194 | **MT035991** | **MT041324** | **MT036179** | **MT041557** | **MT041441** |
| A_K_UFMG9353 | *A. marmorata* | K | UFMG9353 | RJ | Itatiaia, Pousada Chalés Terra Nova | – 22.4565 | – 44.585194 | –– | **MT041325** | **MT036180** | **MT041558** | **MT041442** |
| A_K_UFMG9354 | *A. marmorata* | K | UFMG9354 | RJ | Itatiaia, Estação Ferroviária Campo Belo | – 22.483333 | – 44.564156 | –– | **MT041326** | **MT036181** | **MT041559** | **MT041443** |
| A_L_MTR20994 | *A.* sp.L |  |  | BA | Igrapiuna | – 13.82141 | – 39.136162 | KF675186 | KF675113 | KF674804 | KF674492 | KF674172 |
| A_L_MTR21951 | *A.* sp.L |  |  | BA | E.E..E Wenceslau Guimarães | – 13.69285 | – 39.503775 | –– | KF675114 | KF674805 | KF674493 | KF674173 |
| A_lutzi_ROM40167 | *A. lutzi* |  |  | GUY | Potaro river | 5.166.784 | – 59.782069 | KC603952 | KF674993 | KF674684 | KF674367 | KF674052 |
| A_lutzi_ROM43842 | *A. lutzi* |  |  | GUY | Mt. Wokomung | 5.082.921 | – 59.848759 | –– | KF674994 | KF674685 | KF674368 | KF674053 |
| A_M_MD2568 | *A.* sp.M |  |  | BA | Una | – 15.27281 | – 39.070487 | KF675183 | KF675099 | KF674790 | KF674477 | KF674158 |
| A_M_MTR16429 | *A.* sp.M |  |  | BA | Serra das Lontras, Aratáca | – 15.33611 | – 39.426389 | –– | KF675100 | KF674791 | KF674478 | KF674159 |
| A_marmorata_AF467 | *A. marmorata* | marmorata |  | SP | Ubatuba, Praia Vermelha | – 23.42388 | – 45.046388 | –– | KF675092 | KF674783 | KF674470 | KF674151 |
| A_marmorata_AF906 | *A. marmorata* | marmorata |  | SP | Santa Branca | – 23.4375 | – 45.9375 | –– | KF675093 | KF674784 | KF674471 | KF674152 |
| A_marmorata_CFBH11512 | *A. marmorata* | marmorata | CFBH11512 | SP | Santa Branca | – 23.398552 | – 45.865898 | –– | **MT041327** | **MT036182** | **MT041560** | **MT041444** |
| A_marmorata_CFBH11532 | *A. marmorata* | marmorata | CFBH11532 | SP | Santa Branca | – 23.398552 | – 45.865898 | –– | **MT041328** | **MT036183** | **MT041561** | **MT041445** |
| A_marmorata_CFBH11533 | *A. marmorata* | marmorata | CFBH11533 | SP | Santa Branca | – 23.398552 | – 45.865898 | –– | **MT041329** | **MT036184** | **MT041562** | **MT041446** |
| A_marmorata_CFBH11534 | *A. marmorata* | marmorata | CFBH11534 | SP | Santa Branca | – 23.398552 | – 45.865898 | –– | **MT041330** | **MT036185** | –– | **MT041447** |
| A_marmorata_CFBH11535 | *A. marmorata* | marmorata | CFBH11535 | SP | Santa Branca | – 23.398552 | – 45.865898 | –– | **MT041331** | **MT036186** | **MT041563** | **MT041448** |
| A_marmorata_CFBH11536 | *A. marmorata* | marmorata | CFBH11536 | SP | Santa Branca | – 23.398552 | – 45.865898 | –– | **MT041332** | **MT036187** | **MT041564** | **MT041449** |
| A_marmorata_CFBH12850 | *A. marmorata* | marmorata | CFBH12850 | RJ | Parque Nacional da Tijuca | – 22.96119 | – 43.238611 | KF675180 | KF675087 | KF674778 | KF674465 | KF674146 |
| A_marmorata_CFBH12864 | *A. marmorata* | marmorata | CFBH12864 | SP | Ubatuba, Ilha dos Porcos Pequena | – 23.549471 | – 45.067246 | –– | **MT041333** | **MT036188** | **MT041565** | **MT041450** |
| A_marmorata_CFBH15402 | *A. marmorata* | marmorata | CFBH15402 | SP | Ilha de São Sebastião, Ilhabela | – 23.849569 | – 45.366478 | –– | **MT041334** | **MT036189** | **MT041566** | **MT041451** |
| A_marmorata_CFBH15409 | *A. marmorata* | marmorata | CFBH15409 | SP | Ilha de São Sebastião, Ilhabela | – 23.849569 | – 45.366478 | –– | **MT041335** | **MT036190** | **MT041567** | **MT041452** |
| A_marmorata_CFBH15416 | *A. marmorata* | marmorata | CFBH15416 | SP | Ilha de São Sebastião, Ilhabela | – 23.849569 | – 45.366478 | **MT035992** | **MT041336** | **MT036191** | **MT041568** | –– |
| A_marmorata_CFBH15434 | *A. marmorata* | marmorata | CFBH15434 | SP | Ilha de São Sebastião, Ilhabela | – 23.849569 | – 45.366478 | –– | **MT041337** | **MT036192** | **MT041569** | **MT041453** |
| A_marmorata_CFBH15443 | *A. marmorata* | marmorata | CFBH15443 | SP | Ilha de São Sebastião, Ilhabela | – 23.849569 | – 45.366478 | –– | **MT041338** | **MT036193** | **MT041570** | **MT041454** |
| A_marmorata_CFBH15494 | *A. marmorata* | marmorata | CFBH15494 | SP | Ilha de São Sebastião, Ilhabela | – 23.849569 | – 45.366478 | **MT035993** | **MT041339** | **MT036194** | **MT041571** | –– |
| A_marmorata_CFBH15497 | *A. marmorata* | marmorata | CFBH15497 | SP | Ilha de São Sebastião, Ilhabela | – 23.849569 | – 45.366478 | –– | **MT041340** | **MT036195** | **MT041572** | –– |
| A_marmorata_CFBH15500 | *A. marmorata* | marmorata | CFBH15500 | SP | Ilha de São Sebastião, Ilhabela | – 23.849569 | – 45.366478 | –– | **MT041341** | **MT036196** | **MT041573** | **MT041455** |
| A_marmorata_CFBH15542 | *A. marmorata* | marmorata | CFBH15542 | SP | Ilha de São Sebastião, Ilhabela | – 23.849569 | – 45.366478 | **MT035994** | **MT041342** | **MT036197** | **MT041574** | **MT041456** |
| A_marmorata_CFBH17451 | *A. marmorata* | marmorata | CFBH17451 | SP | Ilha de São Sebastião, Ilhabela | – 23.849569 | – 45.366478 | –– | **MT041343** | **MT036198** | **MT041575** | **MT041457** |
| A_marmorata_CFBH17477 | *A. marmorata* | marmorata | CFBH17477 | SP | Ilha de São Sebastião, Ilhabela | – 23.849569 | – 45.366478 | –– | **MT041344** | **MT036199** | **MT041576** | **MT041458** |
| A_marmorata_CFBH23957 | *A. marmorata* | marmorata | CFBH23957 |  | Caieras | – 23.390520 | – 46.717168 | –– | **MT041345** | **MT036200** | **MT041577** | **MT041459** |
| A_marmorata_CFBH27846 | *A. marmorata* | marmorata | CFBH27846 | RJ | Rio de Janeiro, Parque Nacional da Tijuca | – 22.959778 | – 43.276953 | –– | **MT041346** | **MT036201** | **MT041578** | –– |
| A_marmorata_CFBH27847 | *A. marmorata* | marmorata | CFBH27847 | RJ | Rio de Janeiro, Parque Nacional da Tijuca | – 22.959778 | – 43.276953 | **MT035995** | **MT041347** | **MT036202** | **MT041579** | **MT041460** |
| A_marmorata_CFBH27848 | *A. marmorata* | marmorata | CFBH27848 | RJ | Rio de Janeiro, Parque Nacional da Tijuca | – 22.959778 | – 43.276953 | –– | **MT041348** | **MT036203** | **MT041580** | **MT041461** |
| A_marmorata_CFBH27849 | *A. marmorata* | marmorata | CFBH27849 | RJ | Rio de Janeiro, Parque Nacional da Tijuca | – 22.959778 | – 43.276953 | –– | **MT041349** | **MT036204** | **MT041581** | **MT041462** |
| A_marmorata_CFBH27850 | *A. marmorata* | marmorata | CFBH27850 | RJ | Rio de Janeiro, Parque Nacional da Tijuca | – 22.959778 | – 43.276953 | –– | **MT041350** | **MT036205** | **MT041582** | **MT041463** |
| A_marmorata_CFBH32814 | *A. marmorata* | marmorata | CFBH32814 | SP | Ubatuba, Picinguaba | – 23.373032 | – 44.835442 | **MT035996** | **MT041351** | –– | **MT041583** | **MT041464** |
| A_marmorata_CFBH34389 | *A. marmorata* | marmorata | CFBH34389 | RJ | Maricá | – 22.867078 | – 42.697270 | **MT035997** | **MT041352** | **MT036206** | **MT041584** | **MT041465** |
| A_marmorata_CFBH34391 | *A. marmorata* | marmorata | CFBH34391 | RJ | Maricá | – 22.867078 | – 42.697270 | –– | **MT041353** | **MT036207** | **MT041585** | **MT041466** |
| A_marmorata_CFBH34402 | *A. marmorata* | marmorata | CFBH34402 | RJ | Rio de Janeiro, Parque Nacional da Tijuca | – 22.959778 | – 43.276953 | **MT035998** | **MT041354** | **MT036208** | **MT041586** | **MT041467** |
| A_marmorata_CFBH34404 | *A. marmorata* | marmorata | CFBH34404 | RJ | Rio de Janeiro, Parque Nacional da Tijuca | – 22.959778 | – 43.276953 | **MT035999** | **MT041355** | **MT036209** | **MT041587** | **MT041468** |
| A_marmorata_CFBH34411 | *A. marmorata* | marmorata | CFBH34411 | RJ | Maricá, trilha Sítio Verde, Espraiado | – 22.867078 | – 42.697270 | –– | **MT041356** | **MT036210** | **MT041588** | **MT041469** |
| A_marmorata_CFBH36130 | *A. marmorata* | marmorata | CFBH36130 |  | Nazaré Paulista, IPE | – 23.198649 | – 46.349615 | **MT036000** | **MT041357** | **MT036211** | **MT041589** | **MT041470** |
| A_marmorata_CFBH36131 | *A. marmorata* | marmorata | CFBH36131 |  | Nazaré Paulista, IPE | – 23.198649 | – 46.349615 | –– | **MT041358** | **MT036212** | **MT041590** | **MT041471** |
| A_marmorata_CFBH36132 | *A. marmorata* | marmorata | CFBH36132 |  | Nazaré Paulista, IPE | – 23.198649 | – 46.349615 | –– | **MT041359** | **MT036213** | **MT041591** | **MT041472** |
| A_marmorata_CFBH6395 | *A. marmorata* | marmorata | CFBH6395 | SP | Ubatuba, Picinguaba | – 23.373032 | – 44.835442 | –– | **MT041360** | **MT036214** | **MT041592** | **MT041473** |
| A_marmorata_CFBH6396 | *A. marmorata* | marmorata | CFBH6396 | SP | Ubatuba, Picinguaba | – 23.373032 | – 44.835442 | –– | **MT041361** | **MT036215** | **MT041593** | **MT041474** |
| A_marmorata_CFBHt14290 | *A. marmorata* | marmorata | CFBHt14290 | RJ | Guapimirim, C.P. do Rio de Janeiro | – 22.487577 | – 42.915601 | –– | **MT041362** | **MT036216** | **MT041594** | –– |
| A_marmorata_CFBHt14293 | *A. marmorata* | marmorata | CFBHt14293 | RJ | Cachoeiras de Macacu | – 22.452168 | – 42.771022 | **MT036001** | **MT041363** | –– | **MT041595** | **MT041475** |
| A_marmorata_CFBHt14294 | *A. marmorata* | marmorata | CFBHt14294 | RJ | Cachoeiras de Macacu | – 22.452168 | – 42.771022 | –– | **MT041364** | **MT036217** | **MT041596** | –– |
| A_marmorata_CFBHt14295 | *A. marmorata* | marmorata | CFBHt14295 | RJ | Cachoeiras de Macacu | – 22.452168 | – 42.771022 | –– | **MT041365** | –– | **MT041597** | **MT041476** |
| A_marmorata_CFBHt14296 | *A. marmorata* | marmorata | CFBHt14296 | RJ | Cachoeiras de Macacu | – 22.452168 | – 42.771022 | **MT036002** | **MT041366** | **MT036218** | **MT041598** | –– |
| A_marmorata_CFBHt14305 | *A. marmorata* | marmorata | CFBHt14305 | RJ | Cachoeiras de Macacu, R.E. Guapiaçu | – 22.452168 | – 42.771022 | **MT036003** | **MT041367** | **MT036219** | **MT041599** | ––- |
| A_marmorata_CFBHt14306 | *A. marmorata* | marmorata | CFBHt14306 | RJ | Rio de Janeiro, Parque Nacional da Tijuca | – 22.959778 | – 43.276953 | –– | **MT041368** | **MT036220** | **MT041600** | ––- |
| A_marmorata_CFBHt14307 | *A. marmorata* | marmorata | CFBHt14307 | RJ | Rio de Janeiro, Parque Nacional da Tijuca | – 22.959778 | – 43.276953 | **MT036004** | **MT041369** | **MT036221** | **MT041601** | **MT041477** |
| A_marmorata_CFBHt14314 | *A. marmorata* | marmorata | CFBHt14314 | RJ | Rio de Janeiro, Parque Nacional da Tijuca | – 22.959778 | – 43.276953 | –– | **MT041370** | **MT036222** | **MT041602** | –– |
| A_marmorata_CFBHt14596 | *A. marmorata* | marmorata | CFBHt14596 | RJ | Cachoeiras de Macacu | – 22.452168 | – 42.771022 | –– | **MT041371** | **MT036223** | **MT041603** | **MT041478** |
| A_marmorata_CFBHt14597 | *A. marmorata* | marmorata | CFBHt14597 | RJ | Ilha Grande | – 23.166684 | – 44.182314 | –– | **MT041372** | **MT036224** | **MT041604** | **MT041479** |
| A_marmorata_CFBHt3183 | *A. marmorata* | marmorata | CFBHt3183 | SP | Santa Isabel, Reserva Ibirapitanga | – 23.331443 | – 46.246376 | –– | **MT041373** | **MT036225** | –– | **MT041480** |
| A_marmorata_CFBHt7953 | *A. marmorata* | marmorata | CFBHt7953 | SP | Santa Isabel | – 23.331443 | – 46.246376 | –– | **MT041374** | **MT036226** | **MT041605** | **MT041481** |
| A_marmorata_CFBHt8001 | *A. marmorata* | marmorata | CFBHt8001 | SP | Santa Isabel | – 23.331443 | – 46.246376 | –– | **MT041375** | **MT036227** | –– | **MT041482** |
| A_marmorata_CFBHt8006 | *A. marmorata* | marmorata | CFBHt8006 | SP | Santa Isabel, Fazenda Pilões | – 23.331443 | – 46.246376 | –– | **MT041376** | **MT036228** | **MT041606** | **MT041483** |
| A_marmorata_CFBHt8026 | *A. marmorata* | marmorata | CFBHt8026 | SP | Santa Isabel | – 23.331443 | – 46.246376 | –– | **MT041377** | **MT036229** | **MT041607** | **MT041484** |
| A_marmorata_CMRJ1 | *A. marmorata* | marmorata |  | RJ | Parque Nacional da Tijuca | – 22.96645 | – 43.239126 | –– | KF675086 | KF674777 | KF674464 | KF674145 |
| A_marmorata_CTRU130 | *A. marmorata* | marmorata |  | RJ | Ilha Grande, Estrada para Dois Rios | – 23.166684 | – 44.182314 | **MT036005** | **MT041378** | –– | **MT041608** | **MT041485** |
| A_marmorata_CTRU55 | *A. marmorata* | marmorata |  | RJ | Ilha de Itacuruça, Mangaratiba | – 22.942062 | – 43.893026 | –– | **MT041379** | **MT036230** | **MT041609** | **MT041486** |
| A_marmorata_CTRU56 | *A. marmorata* | marmorata |  | RJ | Ilha de Itacuruça, Mangaratiba | – 22.942062 | – 43.893026 | –– | **MT041380** | **MT036231** | **MT041610** | **MT041487** |
| A_marmorata_CTRU57 | *A. marmorata* | marmorata |  | RJ | Itaguaí, Represa de Itigussu | – 22.811777 | – 43.794109 | **MT036006** | **MT041381** | **MT036232** | **MT041611** | **MT041488** |
| A_marmorata_IGAdR2 | *A. marmorata* | marmorata |  | RJ | Angra dos reis | – 22.99311 | – 44.284716 | –– | KF675090 | KF674781 | KF674468 | KF674149 |
| A_marmorata_MNRJ65495 | *A. marmorata* | marmorata | MNRJ65495 | RJ | Ilha Grande | – 23.13448 | – 44.260597 | –– | KF675088 | KF674779 | KF674466 | KF674147 |
| A_marmorata_MNRJ73063 | *A. marmorata* | marmorata | MNRJ73063 | RJ | Maricá | – 22.91159 | – 42.789803 | –– | KF675084 | KF674775 | KF674462 | KF674143 |
| A_marmorata_MNRJ75916 | *A. marmorata* | marmorata | MNRJ75916 | RJ | Saquarema | – 22.86668 | – 42.486305 | –– | KF675085 | KF674776 | KF674463 | KF674144 |
| A_marmorata_MRT9861 | *A. marmorata* | marmorata |  | SP | Mogi das Cruzes | – 23.47222 | – 46.181944 | –– | KF675094 | KF674785 | KF674472 | KF674153 |
| A_marmorata_MTR14086_AF2113 | *A. marmorata* | marmorata |  | SP | Santana do Parnaíba | – 23.44227 | – 46.918602 | –– | KF675091 | KF674782 | KF674469 | KF674150 |
| A_marmorata_MTR22738 | *A. marmorata* | marmorata | MTR22738 | RJ | Xerém | – 22.54918 | – 43.302298 | –– | KF675089 | KF674780 | KF674467 | KF674148 |
| A_marmorata_UFMG10296 | *A. marmorata* | marmorata | UFMG10296 | RJ | Petrópolis | – 22.516672 | – 43.226702 | –– | **MT041382** | **MT036233** | **MT041612** | **MT041489** |
| A_marmorata_UFMG10297 | *A. marmorata* | marmorata | UFMG10297 | RJ | Petrópolis | – 22.516672 | – 43.226702 | **MT036007** | **MT041383** | **MT036234** | **MT041613** | **MT041490** |
| A_marmorata_UFMG10298 | *A. marmorata* | marmorata | UFMG10298 | RJ | Guapimirim, P.N. da Serra dos Órgãos | – 22.497147 | – 43.000530 | –– | **MT041384** | **MT036235** | **MT041614** | **MT041491** |
| A_marmorata_UFMG10300 | *A. marmorata* | marmorata | UFMG10300 | RJ | Guapimirim, P.N. da Serra dos Órgãos | – 22.497147 | – 43.000530 | –– | **MT041385** | **MT036236** | **MT041615** | **MT041492** |
| A_marmorata_UFMG10302 | *A. marmorata* | marmorata | UFMG10302 | RJ | Guapimirim, P.N. da Serra dos Órgãos | – 22.497147 | – 43.000530 | **MT036008** | **MT041386** | **MT036237** | **MT041616** | **MT041493** |
| A_marmorata_UFMG10304 | *A. marmorata* | marmorata | UFMG10304 | RJ | Guapimirim, P.N. da Serra dos Órgãos | – 22.497147 | – 43.000530 | –– | **MT041387** | **MT036238** | **MT041617** | **MT041494** |
| A_marmorata_UNIBAN1986 | *A. marmorata* | marmorata |  | SP | Biritiba Mirim, Piratininga | – 23.56065 | – 46.036949 | –– | KF675095 | KF674786 | KF674473 | KF674154 |
| A_martinezi_CHUNB40218 | *A. martinezi* |  |  | PA | Novo Progresso | – 7.144954 | – 55.379469 | KC603942 | KF675006 | KF674697 | KF674380 | KF674065 |
| A_martinezi_CHUNB40220 | *A. martinezi* |  |  | PA | Novo Progresso | – 7.144954 | – 55.379469 | –– | KF675005 | KF674696 | KF674379 | KF674064 |
| A_N_CFBHt10241 | *A.* sp.N |  | CFBH21302 | PR | Morretes, estrada da Graciosa | – 25.070805 | – 48.869062 | KF675181 | KF675097 | KF674788 | KF674475 | KF674156 |
| A_nana_CFBHT3251 | *A. nana* |  |  | SC | São Bento do Sul | – 26.233626 | – 49.457236 | –– | KF675097 | KF674768 | KF674455 | KF674136 |
| A_nana_MTR18499 | *A. nana* |  |  | SC | Quital Erica 321 rua Tubarao Joinville | – 26.28647 | – 48.852217 | KF675178 | KF675078 | KF674769 | KF674456 | KF674137 |
| A_nana_MTR18505 | *A. nana* |  |  | PR | Morro Grande, Guaratuba | – 25.97130 | – 48.71403 | –– | KF675076 | KF674767 | KF674454 | KF674135 |
| A_O_CFBHt7321 | *A.* sp.O |  | CFBH17943 | SP | Ilha de Cananéia | – 25.028411 | – 47.956417 | KF675182 | KF675098 | KF674789 | KF674476 | KF674157 |
| A_P_AJC2390 | *A.* sp.P |  |  | COL | Vaupes, Comunidad Trubón, rio Vaupes | 1,21 | – 70.619167 | KF675173 | KF675036 | KF674727 | KF674411 | KF674095 |
| A_P_AJC2464 | *A.* sp.P |  |  | COL | Vaupes, Comunidad Puerto Vaupes, | 1,21 | – 70.619167 | –– | KF675035 | KF674726 | KF674410 | KF674094 |
| A_Q_AJC2490 | *A.* sp.Q |  |  | COL | GuainiaLaguna Pavón, caño Naquen | 2.270.833 | – 68.265278 | KF675174 | KF675037 | KF674728 | KF674412 | KF674096 |
| A_R_MTR18496 | *A. kweti* |  |  | SC | Florianópolis, Trilha dos Naufragados | – 27.82361 | – 48.56168 | KF675176 | KF675065 | KF674756 | KF674442 | KF674124 |
| A_R_TRPD9 | *A. kweti* |  |  | SC | Bombinhas | – 27.19158 | – 48.56904 | –– | KF675066 | KF674757 | KF674443 | KF674125 |
| A_S_978425 | *A.* sp.S |  |  | SP | Fazenda Intervales | – 24.18805 | – 48.358055 | –– | KF675068 | KF674759 | KF674445 | KF674127 |
| A_S_AF512 | *A.* sp.S |  |  | SP | Ribeirão Grande | – 24.09916 | – 48.365277 | –– | KF675069 | KF674760 | KF674446 | KF674128 |
| A_S_CFBHt10262 | *A.* sp.S |  | CFBHt10262 | PR | Guaruva, Serra do Araraquara | – 25.939742 | – 48.922326 | –– | KF675071 | KF674762 | KF674448 | KF674130 |
| A_S_CTMZ02241 | *A.* sp.S |  |  | SP | Cedro, Parque Estadual de Jacupiranga | – 24.32972 | – 48.03 | –– | KF675070 | KF674761 | KF674447 | KF674129 |
| A_S_CY855 | *A.* sp.S |  |  | SP | Caboclos, mun. Iporanga | – 24.45282 | – 48.585069 | –– | KF675072 | KF674763 | KF674449 | KF674131 |
| A_S_ITH0585 | *A.* sp.S |  |  | SP | Buri | – 23.8125 | – 48.5625 | KF675177 | KF675067 | KF674758 | KF674444 | KF674126 |
| A_saci_CHUNB49509 | *A. saci* |  |  | GO | Alto Paraíso de Goiás | – 14.12886 | – 47.499075 | –– | KF675004 | KF674695 | KF674378 | KF674063 |
| A_saci_MTR14648 | *A. saci* |  |  | TO | E.E. Serra Geral do Tocantins | – 11.22083 | – 46.885555 | KF675168 | KF675001 | KF674692 | KF674375 | KF674060 |
| A_simonstuarti_APL13097 | *A. simonstuarti* |  |  | AM | Com.Yamado São Gabriel DAC | – 0.155833 | – 67.086111 | –– | KF674895 | KF674585 | KF674266 | KF673953 |
| A_simonstuarti_LSU13787 | *A. simonstuarti* |  |  | AC | Porto Walter | – 8.266389 | – 72.743611 | KF675166 | KF674885 | KF674575 | KF674256 | KF673943 |
| A_T_MHNC8385 | *A.* sp.T |  |  | FG | Nouragues2 | 40.916.666 | – 52.7 | KF675164 | KF675009 | KF674700 | KF674383 | KF674068 |
| A_thomei_CFBH10573 | *A. thomei* |  | CFBH 22295 | ES | Mimoso do Sul | – 21.070805 | – 41.394715 | KC603946 | KC603971 | KC603999 | KC604043 | KC604067 |
| A_thomei_CTRU22 | *A. thomei* |  |  | RJ | Rio das Ostras, Fazenda das Garças | – 22.502176 | – 41.943694 | –– | KF675101 | KF674792 | KF674479 | KF674160 |
| A_thomei_UFMG9355 | *A. thomei* |  | UFMG9355 | RJ | Itatiaia, Estação Ferroviária Campo Belo | – 22.483333 | – 44.564156 | –– | **MT041388** | **MT036239** | **MT041618** | **MT041495** |
| A_thomei_UFMG9356 | *A. thomei* |  | UFMG9356 | RJ | Itatiaia, Estação Ferroviária Campo Belo | – 22.483333 | – 44.564156 | **MT036009** | **MT041389** | **MT036240** | **MT041619** | **MT041496** |
| H_caparu | *Hidrolaetare caparu* |  |  |  |  |  |  | KC603954 | KC603975 | KC604050 | KC604031 | KC604050 |
| L_rhodomystax | *Leptodactylus rhodomystax* |  |  |  |  |  |  | EU201123 | KC603980 | KC603993 | KC604030 | KC604059 |
| L_lineatus | *Lithodythes lineatus* |  |  |  |  |  |  | EU201136 | JQ321833 | KC604003 | KC604025 | KC604060 |
