# Supplementary material for: One step beyond a broad molecular phylogenetic analysis: Species delimitation of Adenomera marmorata Steindachner, 1867 (Anura: Leptodactylidae)
Source: PLoS One. 2020 Feb 21;15(2):e0229324. doi: 10.1371/journal.pone.0229324 (PMC7034910; doi:10.1371/journal.pone.0229324)
Supplement: S2 Table — Calls were recorded from the Brazilian Atlantic Forest. (DOCX) [file pone.0229324.s004.docx]

**SUPPORTING INFORMATION**

**One step beyond a broad molecular phylogenetic analysis: Species delimitation of *Adenomera marmorata* Steindachner, 1867 (Anura: Leptodactylidae)**

Carla S. Cassini, Pedro P. G. Taucce, Thiago R. de Carvalho; Antoine Fouquet, Mirco Solé, Célio F. B. Haddad and Paulo C. A. Garcia

*Plos One*

**S2 Table. Information associated with analyzed sound files of the six lineages within *Adenomera marmorata* (see Methods). Calls were recorded from the Brazilian Atlantic Forest.**

| **Sound file** | **Lineage** | **Locality (state)** | **Voucher** | **Date** | **Air (°C)** |
| --- | --- | --- | --- | --- | --- |
| PPGT62–63 | Ma1 | Nazaré Paulista (SP) | CFBH 36131 | 17/Feb/2014 | 23.0 |
| PPGT64 | Ma1 | Nazaré Paulista (SP) | CFBH 36132 | 17/Feb/2014 | 23.0 |
| Tijuca_8 | Ma2 | PARNA da Tijuca (RJ) | CFBH 27850 | 04/Jan/2011 | — |
| Tijuca_CV186 | Ma2 | PARNA da Tijuca (RJ) | CFBH 34403 | 17/Jan/2013 | — |
| Tijuca_CV188 | Ma2 | PARNA da Tijuca (RJ) | CFBH 34404 | 17/Jan/2013 | — |
| SerraAraras | Ma2 | Rod. Pres. Dutra km224 (RJ) | — | 20/Dez/2013 | — |
| PPGT36 | Ma2 | Petrópolis (RJ) | UFMG 10296 | 27/Jan/2012 | 20.0 |
| PPGT37 | Ma2 | Guapimirim (RJ) | — | 29/Jan/2012 | 17.5 |
| PPGT38 | Ma2 | Guapimirim (RJ) | UFMG 10304 | 29/Jan/2012 | 17.5 |
| PPGT39 | Ma2 | Guapimirim (RJ) | — | 29/Jan/2012 | 18.0 |
| Adenomera_marmorataParanapSP1aAAGb | J1 | Paranapiacaba (SP) | — | 17/Jan/2004 | 17.5 |
| Adenomera_marmorataParanapSP2bAAGb | J1 | Paranapiacaba (SP) | AAG-UFU 3033 | 17/Jan/2004 | 17.5 |
| Adenomera_marmorataParanapSP3bAAGb | J1 | Paranapiacaba (SP) | AAG-UFU 3039 | 17/Jan/2004 | 17.5 |
| Paranap5_CSC | J1 | Paranapiacaba (SP) | — | 20/Nov/2011 | 19.0 |
| Paranap6_CSC | J1 | Paranapiacaba (SP) | — | 20/Nov/2011 | 19.0 |
| PPGT26 | J1 | Paranapiacaba (SP) | — | 20/Nov/2011 | 19.0 |
| PPGT27 | J1 | Paranapiacaba (SP) | — | 20/Nov/2011 | 18.0 |
| PPGT28–29 | J1 | Paranapiacaba (SP) | — | 20/Nov/2011 | 18.0 |
| PPGT30 | J1 | Paranapiacaba (SP) | UFMG 9469 | 20/Nov/2011 | 18.0 |
| PPGT31 | J1 | Paranapiacaba (SP) | UFMG 9471 | 20/Nov/2011 | 18.0 |
| PPGT32 | J1 | Paranapiacaba (SP) | — | 20/Nov/2011 | 18.0 |
| PPGT33 | J1 | Paranapiacaba (SP) | — | 21/Nov/2011 | 18.0 |
| PPGT34 | J1 | Paranapiacaba (SP) | — | 21/Nov/2011 | 18.0 |
| PPGT35 | J1 | Paranapiacaba (SP) | — | 21/Nov/2011 | 18.0 |
| Neblinas3 | J2 | Mogi das Cruzes (SP) | BB 311 | 14/Nov/2011 | — |
| Neblinas1 | J2 | Mogi das Cruzes (SP) | BB 296 | 14/Nov/2011 | — |
| Neblinas4 | J2 | Mogi das Cruzes (SP) | BB 319 | 14/Nov/2011 | — |
| Adenomera_marmorataUbatubaSP1bAAGm | K1 | Ubatuba (SP) | — | 29/Oct/2010 | 19.4 |
| PPGT55–56 | K1 | Ubatuba (SP) | — | 21/Nov/2013 | 24.5 |
| PPGT57 | K1 | Ubatuba (SP) | — | 21/Nov/2013 | 24.5 |
| PPGT58 | K1 | Ubatuba (SP) | CFBH 36001 | 21/Nov/2013 | 24.5 |
| PPGT59 | K1 | Ubatuba (SP) | CFBH 36005 | 22/Nov/2013 | — |
| TRC181 | K1 | São Luiz do Paraitinga (SP) | — | 18/Dec/2017 | 23.0 |
| TRC182 | K1 | São Luiz do Paraitinga (SP) | — | 20/Dec/2017 | 21.0 |
| PPGT52 | K1 | São Luiz do Paraitinga (SP) | — | 20/Nov/2013 | 27.0 |
| PPGT53 | K1 | São Luiz do Paraitinga (SP) | CFBH 35996 | 20/Nov/2013 | 22.0 |
| PPGT54 | K1 | São Luiz do Paraitinga (SP) | CFBH 35998 | 20/Nov/2013 | 21.0 |
| PPGT60 | K2 | Muriaé (MG) | CFBH 35983 | 18/Jan/2014 | — |
| PPGT61 | K2 | Muriaé (MG) | — | 18/Jan/2014 | — |
| PPGT40 | K2 | PARNA do Itatiaia (RJ) | UFMG 13283 | 14/Dec/2012 | 19.0 |
| PPGT41 | K2 | PARNA do Itatiaia (RJ) | UFMG 13284 | 14/Dec/2012 | 19.0 |
| PPGT42 | K2 | PARNA do Itatiaia (RJ) | — | 15/Dec/2012 | 20.0 |
| PPGT43 | K2 | PARNA do Itatiaia (RJ) | — | 15/Dec/2012 | 21.0 |
| PPGT44 | K2 | PARNA do Itatiaia (RJ) | — | 15/Dec/2012 | 21.0 |
| PPGT45 | K2 | PARNA do Itatiaia (RJ) | UFMG 13285 | 15/Dec/2012 | 21.0 |
| PPGT46 | K2 | PARNA do Itatiaia (RJ) | — | 15/Dec/2012 | 21.0 |
| PPGT47 | K2 | PARNA do Itatiaia (RJ) | — | 15/Dec/2012 | 20.0 |
| PPGT48 | K2 | PARNA do Itatiaia (RJ) | — | 15/Dec/2012 | 20.0 |
| PPGT49 | K2 | PARNA do Itatiaia (RJ) | UFMG 13289 | 16/Dec/2012 | 17.0 |
| PPGT50 | K2 | PARNA do Itatiaia (RJ) | — | 16/Dec/2012 | 17.0 |
| PPGT51 | K2 | PARNA do Itatiaia (RJ) | UFMG 13291 | 16/Dec/2012 | 21.0 |
