# Supplementary material for: One step beyond a broad molecular phylogenetic analysis: Species delimitation of Adenomera marmorata Steindachner, 1867 (Anura: Leptodactylidae)
Source: PLoS One. 2020 Feb 21;15(2):e0229324. doi: 10.1371/journal.pone.0229324 (PMC7034910; doi:10.1371/journal.pone.0229324)
Supplement: S3 Table — Temporal traits were obtained from waveforms; spectral traits from spectrograms and amplitude spectra. RMS = root mean square. (DOCX) [file pone.0229324.s005.docx]

**SUPPORTING INFORMATION**

**One step beyond a broad molecular phylogenetic analysis: Species delimitation of *Adenomera marmorata* Steindachner, 1867 (Anura: Leptodactylidae)**

Carla S. Cassini, Pedro P. G. Taucce, Thiago R. de Carvalho; Antoine Fouquet, Mirco Solé, Célio F. B. Haddad and Paulo C. A. Garcia

*Plos One*

**S3 Table. Acoustic terminology and definitions for the automated analysis of acoustic traits.** Temporal traits were obtained from waveforms; spectral traits from spectrograms and amplitude spectra. RMS = root mean square.

| **terminology** | **Definition** |
| --- | --- |
| **Temporal traits** |  |
| Call rate | (Calls - 1) / length between the onset of first and last calls |
| Call length | From first 10% amplitude to final 10% amplitude |
| Call rise time | Time of maximum amplitude |
| Call attack length | From first 10% to 90% amplitude |
| Call decay length | From final 90% amplitude to final 10% |
| Call plateau length | From first 90% amplitude to final 90% amplitude |
| Call attack shape (unitless) | Amplitude ratio between first 10–50% and 10–90% |
| Call decay shape (unitless) | Amplitude ratio between final 90–50% and 90–10% |
| Crest factor (unitless) | Ratio between peak amplitude and RMS of the call* |
| **Spectral traits** |  |
| Call dominant frequency | Frequency containing the greatest energy in the call |
| Call frequency modulation | Final 90% minus first 90% amplitude (peak frequencies) |
| Call fundamental frequency | Peak frequency in the first harmonic |
| Call attack frequency | Peak frequency contained in the first 90% amplitude |
| Call decay frequency | Peak frequency contained in the final 90% amplitude |
| Call Bandwidth | At 10% peak amplitude (-20 dB_SPL_); in the dominant harmonic |
